# Variable Effects of Twenty Sugars and Sugar Alcohols on the Retrogradation of Wheat Starch Gels

**DOI:** 10.3390/foods11193008

**Published:** 2022-09-27

**Authors:** Matthew C. Allan, Lisa J. Mauer

**Affiliations:** 1 Department of Food Science, Purdue University, 745 Agriculture Mall Drive, West Lafayette, IN 47907, USA; 2 USDA-ARS, SEA, Food Science and Market Quality and Handling Research Unit, 322 Schaub Hall, North Carolina State University, Raleigh, NC 27695, USA

**Keywords:** starch, retrogradation, sweetener stereochemistry, molar volume, Avrami equation, sugar, sugar alcohol

## Abstract

Starch retrogradation is desirable for some food textures and nutritional traits but detrimental to sensory and storage qualities of other foods. The objective of this study was to determine the impact of sweetener structure and concentration on the retrogradation of wheat starch gels. The effects of 20 sweeteners selected based on common food usage and stereochemical structures of interest, and ranging in concentration from 10 to 50%*w*/*w*, on the retrogradation of wheat starch gels were monitored spectrophotometrically over time. The sweeteners were sucrose, xylose, ribose, glucose, galactose, fructose, mannose, mannitol, L-sorbose, xylitol, tagatose, allulose, maltose, lactose, isomaltulose, isomalt, sorbitol, maltitol, and raffinose. Retrogradation rates and amounts were compared by Avrami equation rate constants (*k* = 0.1–0.7) and absorbance values measured on day 28 (Abs = 0.1–1.0), respectively. Both sweetener concentration and type significantly affected retrogradation. Gels made with sugar alcohols and high sweetener concentrations (≈≥40%) tended to retrograde more and faster, whereas gels made with sugars and low sweetener concentrations tended to have lower retrogradation rates and amounts. Sweeteners with more equatorial and exocyclic hydroxyl groups (e.g., glucose and maltitol) and those with larger molar volumes (e.g., isomaltulose and raffinose) tended to increase the rate and amount of retrogradation, particularly at higher concentrations. The impact of sweeteners on retrogradation was a balance of factors that promoted retrogradation (intermolecular interactions and residual short-range molecular order) and inhibiting behaviors (interference at crystallization sites), which are influenced by sweetener concentration and structure. Understanding which sweeteners at which concentrations can be used to promote or inhibit retrogradation is useful for product formulation strategies.

## 1. Introduction

Starch granules are semicrystalline biological structures largely composed of two α-glucans: amylose and amylopectin. Native starch granules are water insoluble and relatively inert in foods, but in the presence of water (or other low molecular weight plasticizers such as glycerol or sugar solutions) and sufficient heat to reach the gelatinization temperature, the crystalline regions of the starch granule melt and there is an irreversible loss of the native structure [1,2]. During gelatinization and pasting, starch granules imbibe more of the surrounding solution, swell to several times their original size, and amylose begins to leach out; with continued heating and mixing, the granules eventually rupture, releasing their remaining contents [2]. The type and concentration of sweetener in solution elevate the gelatinization temperature (T_gel_) of starch due to the formation of stabilizing hydrogen bonds between sweeteners and starch polymers in the amorphous regions of the starch granules and reduction in the volume density of hydrogen bonding sites with corresponding reduced plasticizing ability of the solvent compared to water [3,4].

Upon cooling after gelatinization, amylose and amylopectin begin to retrograde, reassociating via chain entanglements that lead to the formation of ordered crystalline regions [5]. The rate and extent of amylose and amylopectin retrogradation differ. Amylose crystallizes in minutes to hours and contributes to starch gel formation as well as the setting of bread [6]. Amylose can crystallize either as a double helix or as a single helical V-type crystal with a less polar molecule (e.g., fatty acid, alcohol, surfactant) in the hydrophobic center, and both of these crystal types have a relatively high melting temperature at ≈120 °C [5]. Amylopectin retrogrades more slowly (days) and has a lower melting temperature range, from ≈35 to 80 °C depending on the storage conditions [7]. Amylopectin retrogrades into double helical B-type crystals but, at higher temperatures (>55 °C), can crystallize into the A-type or C-type (mixture of A and B-type crystals) [5]. The external amylopectin chains may also form some single helix V-type crystalline regions with lipids [5]. Amylopectin crystallization may be undesirable in some food products, leading to syneresis, decreased gel clarity, increased gel stiffness, and bread staling, but desirable in other products including the production of resistant starch [2,6,8]. 

The rate and amount of retrogradation are dependent on many factors such as the botanical source and starch fine structure [8,9,10], relative humidity and moisture content (i.e., glass transition temperature (T_g_) of amorphous fraction) [11,12], storage temperature [13,14], heating conditions (e.g., cooking time and temperature) [15,16], starch–lipid interactions [17,18], and amylase hydrolysis of the starch structure [19]. Thus, both intrinsic and extrinsic factors affect starch retrogradation. More details on the effects of these factors on retrogradation have been extensively reported in reviews such as by Wang et al. [20], Hoover [21], Fu et al. [22], and Zobel and Kulp [5]. A recent study has also explored the relationship of the amount of residual short-range molecular order in gelatinized wheat starch to the subsequent retrogradation [23].

The effects of sweeteners (sugars and sugar alcohols) on starch retrogradation have also been investigated; however, the findings are contradictory. For example, sweeteners were reported to promote the retrogradation of oat [24], normal corn [25], normal, high amylose, and waxy corn [18], and potato starches [26], whereas, sweeteners were also reported to suppress retrogradation of wheat [27], oat and wheat [28], tapioca [29], normal and waxy corn [30], and rice starches [31,32,33]. Depending on additional experimental variables (sweetener type, concentrations, storage temperatures), sweeteners were also reported to both increase and decrease retrogradation of wheat [26,34], waxy corn, wheat, potato and pea [35], rice [36], waxy corn [37,38], waxy and normal corn [39], *Pueraria lobata* (kudzu) [40], and amaranth starches [41]. These contradictory findings demonstrate the complexity of the effects of different sweeteners on starch retrogradation and highlight the need for a better understanding of what sweetener traits are associated with their effects on starch retrogradation. Therefore, the objective of this study was to compare the effects of 20 different sweeteners across a wide range of concentrations on the retrogradation of wheat starch gels to elucidate which sweetener properties are responsible for promoting or inhibiting starch retrogradation. This will provide foundational information and aid in the development of food formulation strategies to control starch retrogradation, and/or reduce or replace conventional sugars.

## 2. Materials and Methods

### 2.1. Materials

Aytex^®^ P wheat starch, an unmodified, highly purified native wheat starch (<0.2% protein, <0.1% fat, <0.2% ash, 9.9% water, and 25% amylose) [42] was donated by ADM (Minneapolis, MN, USA) and used “as is”. Twenty different sugars and sugar alcohols that may be found in food products and/or have stereochemical structures of interest were used: xylose, ribose, glucose, galactose, fructose, mannose, and mannitol from Acros Organics (Fair Lawn, NJ, USA); L-sorbose and xylitol from Sigma-Aldrich (St. Louis, MO, USA); trehalose dihydrate from Hayashibara Company (Okayama, JP, USA); tagatose and allulose from Sensato (Albany, NY, USA); maltose monohydrate and lactose monohydrate from Fisher Chemical (Fair Lawn, NJ, USA); isomaltulose monohydrate and isomalt ST (~1:1 ratio of glucopyranosyl sorbitol and glucopyranosyl mannitol dihydrate [43]) from BENEO-Palatinit Gmbh (Mannheim, DE, USA); sorbitol from Amresco (Solon, OH, USA); sucrose from Mallinckrodt Chemicals (Phillipsburg, NJ, USA); and maltitol and raffinose pentahydrate from Alfa Aesar (Ward Hill, MA, USA) (Table 1). Calcium propionate was from Sigma-Aldrich. The water used in this study was processed using reverse osmosis, then filtered by a Barnstead E-Pure Lab Water System (Dubuque, IA, USA) to >17.4 milliohm-cm. 

### 2.2. Methods

#### 2.2.1. Sweetener Solution Preparation

Sweetener solutions at 10, 20, 30, 40, and 50%*w*/*w* (when possible based on sweetener solubility limits) were prepared in 10 mL volumes in 15 mL centrifuge tubes and stored at −20 °C when not in use for extended periods. The solubility limits for mannitol and lactose prevented production of the higher solution concentrations. Moisture contents of the sweeteners that were crystal hydrates were accounted for when preparing solutions. To defrost and encourage sweetener dissolution, solutions were heated at 60–80 °C in a heating block for 5–15 min, followed by rotational mixing on a Scientific Industries Roto-Shake Genie (Bohemia, NY, USA). This was repeated until the sweetener was fully dissolved. Solutions were not used if crystals were visibly present after overnight storage at ambient conditions.

#### 2.2.2. Spectrophotometric Absorbance Measurements

The retrogradation measurement method using spectrophotometric absorbance values of starch gels was adapted from Jacobson et al. [52]. An advantage to using spectrophotometry for monitoring retrogradation is that the measurements are repeated for the same sample throughout the experiment since the method is non-destructive, which eliminates sample-to-sample variability between timepoints and more efficient throughput than more destructive analyses such as differential scanning calorimetry. Wheat starch-sweetener slurries were prepared in 1.5 mL centrifuge tubes using 0.1 g wheat starch, 1 mL sweetener solution (of 20 sweetener types ranging in concentration from 10 to 50%*w*/*w*), and 4 μL of a 25%*w*/*w* calcium propionate solution (preservative). Slurries were made on a starch weight-to-solution volume ratio to achieve similar gelatinized starch volumes in each gel. Slurries were vortexed until fully mixed, immediately poured into the well of a folded hexagon polystyrene weigh dish (4.7 cm inner diameter), then 40 μL of slurry was pipetted into each of 6 wells of a clear polystyrene flat bottomed 96-well plate using a 6 channel pipettor. The plates were sealed with a thermal adhesive sealing film for polymerase chain reaction plates and heated for 90 min at 99 °C (to gelatinize the starch) in a digital AccuTemp-09s vacuum oven (Across Instruments, Livingston, NJ, USA) at ambient pressure. Preheated metal blocks were also placed on top of each plate to maintain the seal and for better heat transfer. After heating, the plates were held at ambient temperature for 1 h to allow for the initial amylose retrogradation and vapor condensation inside the wells to take place. Water that condensed on the inside of the film was knocked back into the wells by gently tapping the plate to maintain the original moisture contents. The plates were then loaded into a Beckman Coulter AD 340 (Brea, CA, USA) Microplate Reader, and the absorbance values at 620 nm were measured (day 0). Plates were resealed with a new adhesive sealing film and stored at 4 °C. Absorbances were measured on days 1, 3, 7, 14, 21, and 28, and plates were resealed with a new adhesive sealing film after each absorbance measurement. The controls were starch with water and water alone. 

#### 2.2.3. Differential Scanning Calorimetry

The gelatinization temperatures of the starch-sweetener slurries across all sweetener concentrations were determined using differential scanning calorimetry, as described by Allan, Rajwa, and Mauer [3]. Additionally, sucrose-starch gels made using 0, 10, 20, 30, 40, and 50% sucrose solutions were prepared, ~10 mg of each slurry (precise weight was recorded) was pipetted into a Perkin Elmer 50 µL differential scanning calorimeter (DSC) pan (BO143017), and each pan was hermetically sealed with a lid (BO143003). Six replicates were made for each sucrose concentration. These sealed pans were placed in a 96-well plate and held at 99 °C for 90 min in an AccuTemp-09s vacuum oven followed by storage at 4 °C, replicating the conditions used for the absorbance measurement studies. Of the 6 replicates for each sucrose concentration, 3 replicates were analyzed after 7 days and the other 3 after 14 days of incubation at 4 °C. The enthalpy of retrogradation (ΔH) was measured by manually transferring pans into a Perkin Elmer DSC 4000 (Waltham, MA) that was calibrated using water, indium, and zinc. Each sample was heated from 30 to 100 °C at 10 °C/min. The retrogradation peak in the thermogram was identified as the single endothermic event that occurred between 40 and 60 °C. An additional set of sucrose-starch slurries containing 0, 10, 20, 30, 40, and 50% sucrose solutions were prepared for determining starch gelatinization properties. The sweetener solutions and starch were combined, ~10 mg was sealed into a DSC pan, the pans were transferred into the DSC, and each sample was heated from 30 to 100 °C at 10 °C/min. The regions of the thermogram from 2 to 5 °C before and after the end of the endothermic peaks were used to determine the onset temperature, peak temperature, and ∆H of retrogradation and gelatinization endotherms by the “peak calculation” function with the “Standard” baseline in Pyris Software (version 10.1.0.0412). The ∆Hs of retrogradation and gelatinization were calculated as the area of the peak, and the reported J/g is the enthalpy of the slurry. 

#### 2.2.4. Data Analysis

A modified Avrami equation adapted from Berski, Ziobro, Witczak, and Gambus [28] was used to model and compare the rate constants of retrogradation:

(1) $${\mathrm{Abs}}_{t}={\mathrm{Abs}}_{\infty }-\left({\mathrm{Abs}}_{\infty }-{\mathrm{Abs}}_{0}\right)*e^{-kt^{n}}$$

where Abs*_t_* was the predicted absorbance at time *t*, Abs_∞_ was the absorbance at day 28, *k* was the calculated rate constant, *t* was days of incubation, and *n* was the Avrami exponent. Rate constants (*k*) of retrogradation were calculated using absorbance measurements and the Curve Fitting Application in MatLab R2019a (MathWorks Inc., Natick, MA, USA) for the 620 nm absorbances (*y*-axis) plotted with respect to time (days, *x*-axis). The Avrami exponent (*n*) was set to 1 to compare retrogradation rate constants (*k*) despite the slightly weaker fit than with a free *n* variable in Equation (1). The Avrami exponent (*n*) for starch retrogradation has been previously calculated to be 1, which assumes that starch crystallization was “rod-like growth from an instantaneous nuclei” [53]. Equation (1) and the calculated *k* values were used to model the absorbances of the starch gels. In addition to determining rate constants (*k*), the amount of retrogradation was documented by using Abs_∞_ (absorbance at day 28) and the change of absorbance from day 0 to day 28 (Abs_(∞-0)_) to offset for initial absorbance differences. 

The effects of categorical sweetener solution properties (number of carbons in a sweetener, sugar or sugar alcohol, reducing sugar, and sweetener concentration (Table 1)) on retrogradation properties (*k*, Abs_∞_, and Abs_(∞-0)_) were analyzed using 2-way ANOVA (α = 0.05) in the Standard Least Squares “personality” in JMP Pro 14.0.0 (SAS Institute Inc., Cary, NC, USA). Statistical differences between the onset temperatures, peak temperatures, and enthalpies measured by DSC were compared by one-way ANOVA with a Tukey post hoc test (α = 0.05) in JMP Pro 14.0.0.

The molar effective number of hydroxyl groups (*N_OH,eff_*) and effective volume fraction of the solvent ϕ_w,eff_ of sweetener solutions were calculated as described in van der Sman and Mauer [4] using values from Table 1. The effects of numerical sweetener properties (number of equatorial and exocyclic hydroxyl groups, dry T_g_, molar volumes (Table 1), *N_OH,eff_*, and ϕ_w,eff_ (Table S3)), as well as onset T_gel_, on retrogradation were investigated by a series of linear correlations. Retrogradation rate constants (*k*) and amounts (Abs_∞_, Abs_(∞-0)_) for the sweetener-starch gels were grouped by sweetener concentration, then individual Pearson correlation coefficients (*r*) were calculated between the retrogradation properties (*y*-axis) against the numerical sweetener properties (*x*-axis). For example, *r* was calculated for the linear correlation between the *k* values of gels made with 10% sweetener solutions against the molar volumes of the sweeteners. Significances of *r* values were assessed using two-tailed *t*-statistics with Microsoft Excel 365 (Redmond, WA, USA).

## 3. Results and Discussion

### 3.1. Sucrose Concentration Effects on Retrogradation of Wheat Starch Gels

**Figure 1:**
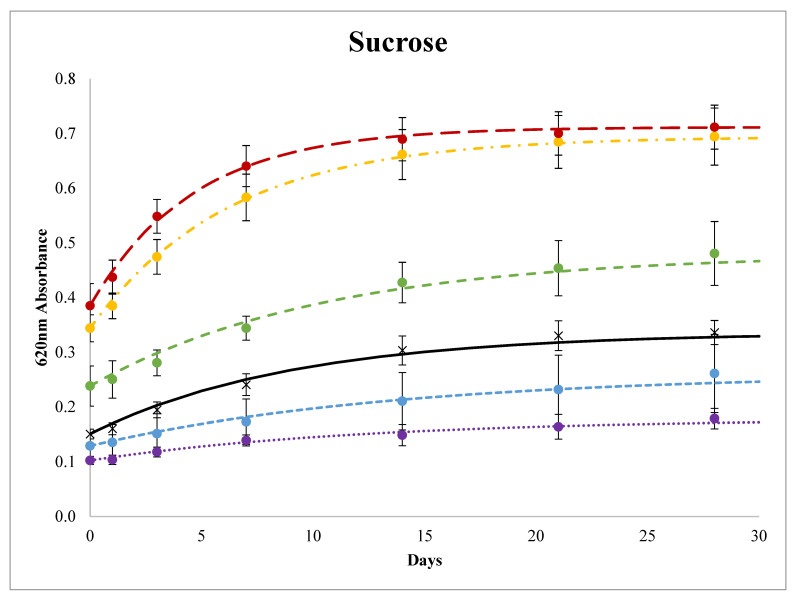
Changes in 620 nm absorbance over 28 days at 4 °C of 10%*w*/*v* wheat starch gels made with water (black, ×──), 10% (purple, ●∙∙∙∙∙), 20% (blue, ●----), 30% (green, ●- - -), 40% (yellow, ●- ∙ - ∙), 50% (red, ●― ―) *w*/*w* sucrose solutions. Lines are Avrami modeled absorbance values and the error bars are 1 standard deviation of the measured absorbance values.

Wheat starch gels made with 0 to 50% sucrose solutions exhibited increasing Abs_620nm_ values over time, and the initial absorbance values (day 0) were greater with increasing sucrose concentrations (Figure 1). The Abs_620nm_ values of the sweetener solutions without starch were ≈0.0; thus, the sweetener solutions themselves did not contribute to the absorbance values. Differences in the initial starch gel Abs_620nm_ values were likely from varying degrees of granule swelling and extents of amylose retrogradation because initial starch gel turbidity is attributed to granule remnants and swollen granules that refract and scatter light [54]. Starch granule swelling likely varied due to the increasing sweetener concentrations increasing both the gelatinization temperature [3] and pasting temperature [55,56]. Increasing sucrose concentrations increased the T_gel_ of starch from 64.3 °C in 10% sucrose solutions to 89.6 °C in 50% solutions (Table 2); therefore, the temperature differential between T_gel_ and the gel preparation conditions (90 min at 99 °C) varied even though all samples were heated above the T_gel_. Wheat starch pasting temperatures in 43% (1.5M) and 55% (2M) sucrose solutions are less than 95 °C, but the starch in these solutions does not fully swell as indicated by lowered peak pasting viscosities with no viscosity breakdown [56]. Thus, the samples in Figure 1 would have all gelatinized and begun pasting, but more residual short-range molecular order and less granule swelling likely occurred as sucrose concentrations increased which contributed to higher initial Abs_620nm_ values. 

**Figure 2:**
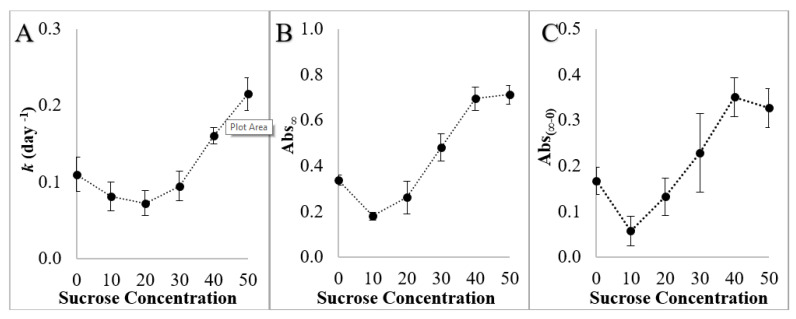
(**A**) Retrogradation rate constants (*k*), (**B**) absorbances at day 28 (Abs_∞_), (**C**) change in absorbance values from day 0 to day 28 (Abs_(∞-0)_) of starch gels made with 0–50% sucrose solutions. Error bars are 95% confidence intervals for retrogradation rates (**A**) and 1 standard deviation for absorbance values (**B**,**C**).

Increases in absorbance over time are attributed to amylopectin retrogradation. As starch retrogrades, intra- and intermolecular starch–starch junction points grow into crystalline zones, resulting in lower starch solubility and dispersed particles large enough to scatter light [54]. The rate and extent of retrogradation was affected by the sucrose concentration: the gels made with 10 and 20% sucrose solutions retrograded less than the control (0% sucrose) and gels made with 30, 40, and 50% sucrose solutions had more retrogradation (Figure 1 and Figure 2). The calculated Avrami rate constant (*k*) decreased with sucrose concentrations up to 20% but then increased with increasing sucrose concentrations in gels made with >30% sucrose solutions (Figure 2). This suggests that increasing sucrose at lower concentrations (0 to 20%) may have a retrogradation antagonistic effect (34% reduction in *k* and 22% reduction in Abs_∞_) but increasing sucrose at higher concentrations will promote retrogradation (≈2 fold increase in *k* and Abs_∞_). In the absence of sugar, Huang, Chao, Yu, Copeland and Wang [23] found that initial increases in water content and heating temperature promoted the retrogradation of wheat starch (attributed to greater glucan chain flexibility upon some decrease in short-range molecular order) but further increases in water content and heating temperature inhibited starch retrogradation (attributed to a weaker nucleation effect from the decreased amount of residual short-range molecular order). The addition of increasing amounts of sucrose in solution decreases the volume fraction of water, stabilizes the amorphous regions of starch, increases the T_gel_, and resulted in increases in retrogradation, which were likely due to enough residual short-range molecular order to serve as nucleation sites and enough molecular mobility of the glucan chains to facilitate retrogradation. However, this does not explain the inhibition of retrogradation at the lower sucrose concentrations compared to the water control.

**Figure 3:**
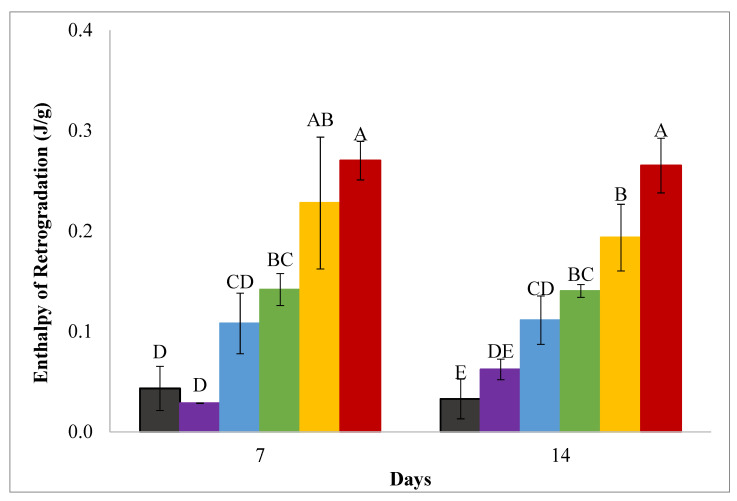
Enthalpies of retrogradation of 10%*w*/*v* wheat starch gels made with (from left to right) water (black), 10% (purple), 20% (blue), 30% (green), 40% (yellow), 50% (red) *w*/*w* sucrose solutions and incubated at 4 °C for 7 and 14 days. Error bars are 1 standard deviation and significant differences are indicated by capital letters (A–E).

The melting enthalpies of wheat starch–sucrose gels determined by DSC analysis of the same sucrose-containing gels analyzed in the spectrophotometer are reported in Figure 3. Consistent with the retrogradation trends determined by monitoring absorbance (Figure 1), the melting enthalpy results indicate that gels made with 30, 40, and 50% sucrose solutions exhibited increased retrogradation after 7 and 14 days (Figure 3), with the most retrogradation occurring at the highest sucrose concentration. The effects of lower sucrose concentrations on retrogradation differed between the DSC and spectrophotometer results in that no inhibition of retrogradation was found in the DSC analyses. Gels made with 10 and 20% sucrose solutions were not different from the control after 7 days, gels made with 20% sucrose had a greater enthalpy of retrogradation after 14 days (Figure 3). Both gel absorbance/turbidity and melting enthalpy values are associated with retrogradation but are measurements of different retrogradation attributes [20]. The enthalpy is the energy to remelt retrograded starch, while absorbance/turbidity measures light scattering from retrograded starch aggregates [20]. The lower amounts of sucrose affected the trends in these two measurements differently.

The melting peak temperatures of retrograded sucrose-starch gels ranged from 55.07 to 60.92 °C, and only gels made with 40 and 50% sucrose had higher melting temperatures than gels made with water (Table S1). Retrograded starch melting temperatures and temperature ranges were much lower than the gelatinization temperatures in 0 to 50% sucrose solutions (Table S1). The lower melting temperatures of retrograded starch were likely due to retrograded starch adopting the B-type polymorph, which has a lower melting temperature than the native wheat starch A-type polymorph [5,57]. 

### 3.2. Effects of 20 Sweeteners at Different Concentrations on the Retrogradation of Wheat Starch-Sweetener Gels

**Figure 4:**
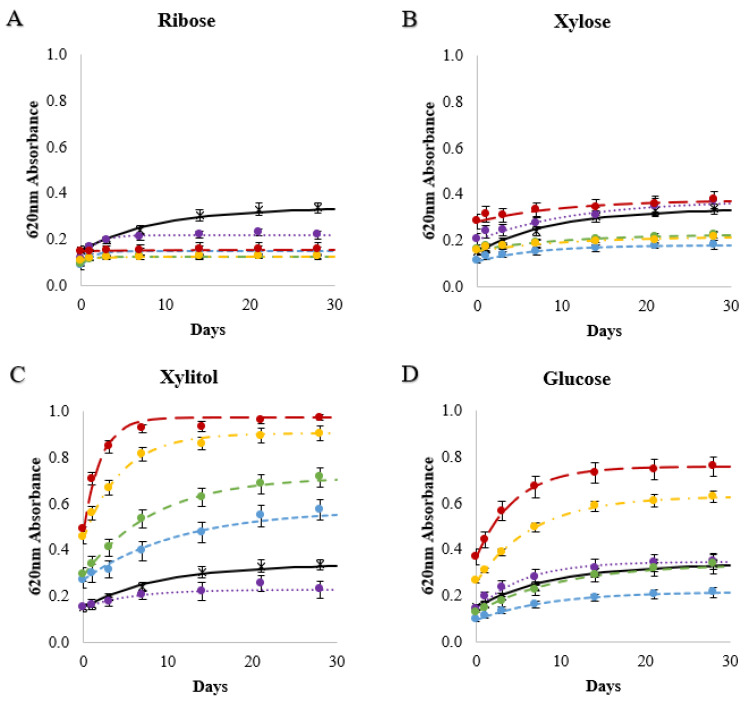

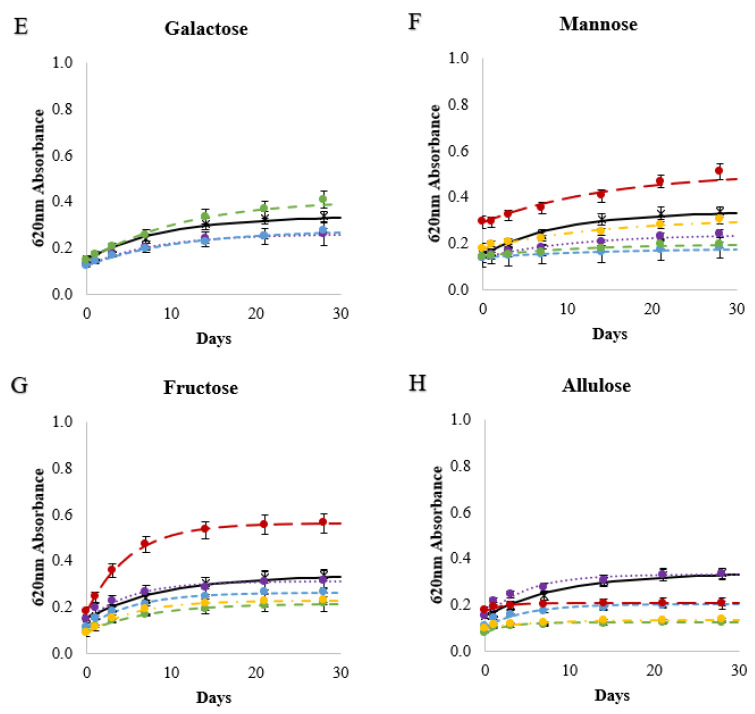

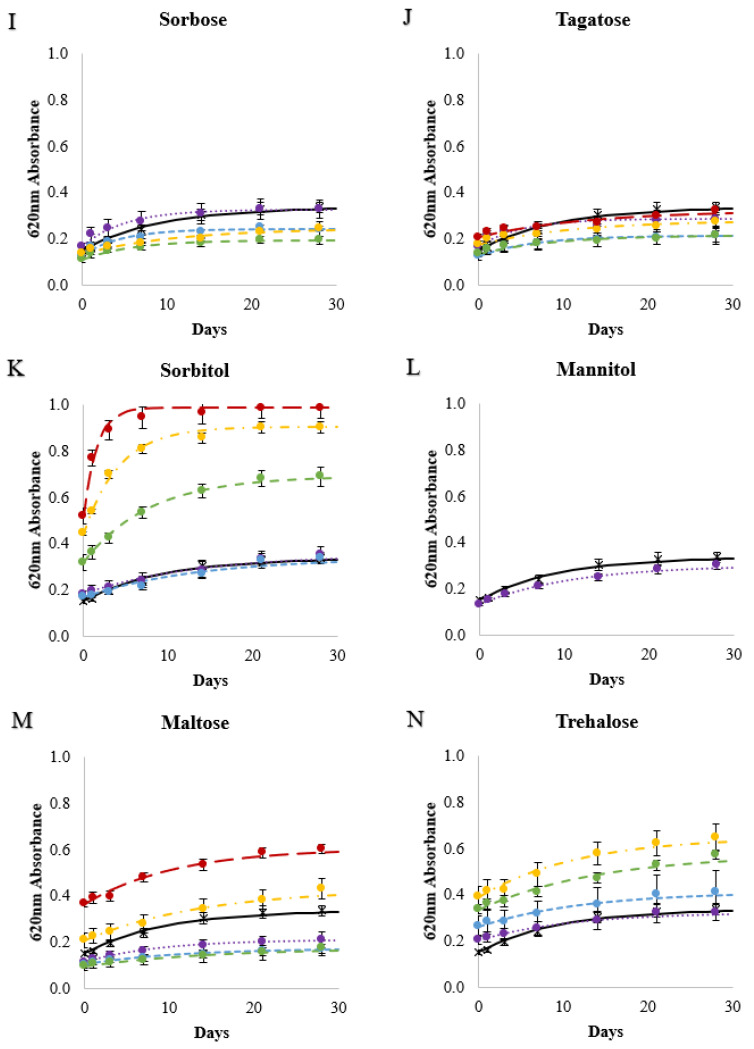

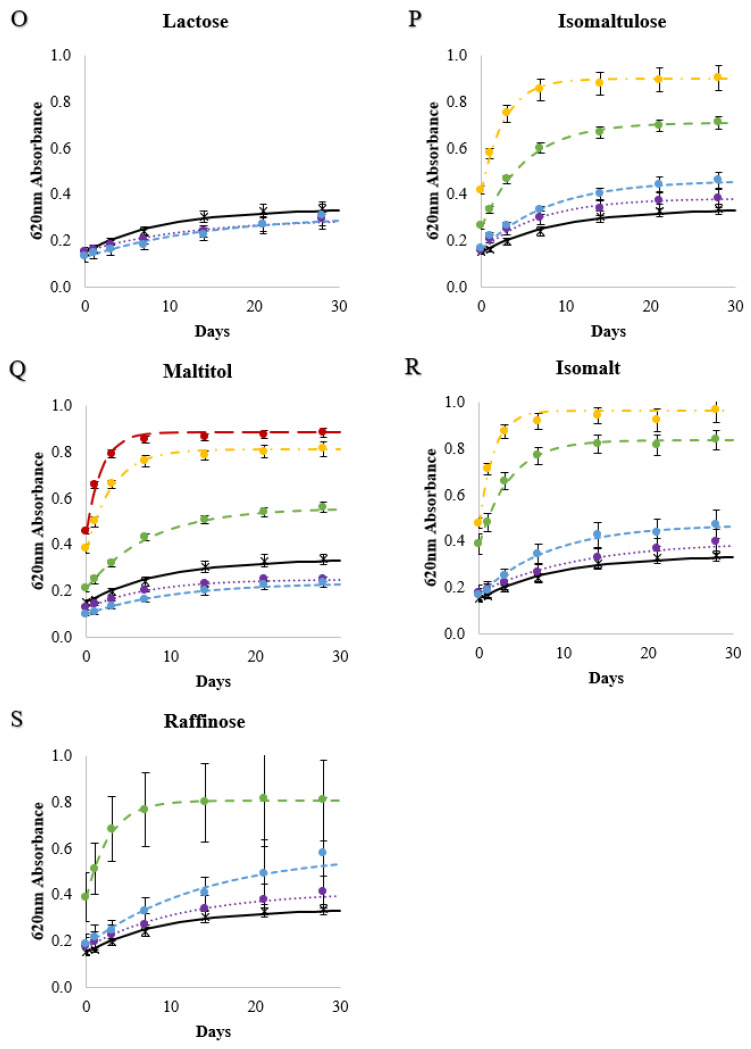
Changes in 620 nm absorbances of 10%*w*/*v* wheat starch gels stored at 4 °C over 28 days made with water (black, ×──), 10% (purple, ●∙∙∙∙∙), 20% (blue, ●----), 30% (green, ●- - -), 40% (yellow, ●- ∙ - ∙), 50% (red, ●― ―) solutions of 5-C sugars (**A**,**B**), a 5-C sugar alcohol (**C**), 6-C sugars (**D**–**J**), 6-C sugar alcohols (**K**,**L**), 12-C sugars (**M**–**P**), 12-C sugar alcohols (**Q**,**R**), and a 18-C sugar (**S**). Lines are Avrami modeled absorbance values and the error bars are 1 standard deviation of the measured absorbance values.

The effects of sweetener solutions (20 sweeteners at 10 to 50%*w*/*w* concentrations) on the retrogradation rate constants (*k*) and amounts (Abs_∞_ and Abs_(∞-0)_) in 10% wheat starch gels were compared (Figure S1). Both sweetener type and concentration were found to significantly affect retrogradation (Table 2), in some cases increasing retrogradation and in others decreasing retrogradation (Figure 4). The control starch gel containing no sweetener was near the median for *k* and absorbance values, at the 43rd, 41st, and 59th percentile from the bottom of *k*, Abs_∞_, and Abs_(∞-0)_ values, respectively. Starch–sweetener gels with the highest rates and amounts of retrogradation, exhibiting *k*, Abs_∞_, and Abs_(∞-0)_ values in the top quartile, were made with 50% glucose, 30 and 40% isomalt, 40% isomaltulose, 40 and 50% maltitol, 30% raffinose, 40 and 50% sorbitol, 50% sucrose, and 40 and 50% xylitol solutions. Gels in which retrogradation was inhibited, for which *k*, Abs_∞_, and Abs_(∞-0)_ were in the lowest quartile, were made with 30% maltose, 20% mannose, and 10% sucrose solutions. 

**Figure 5:**
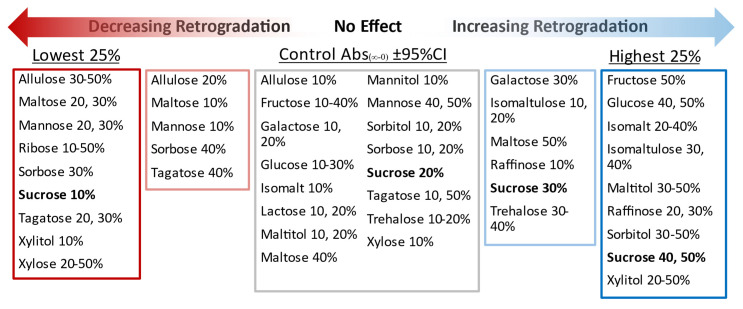
Overview of sweetener solution type and concentration effects on starch retrogradation in wheat starch gels.

Based on the absorbance data (Figure 4), sweeteners were grouped into three general categories: retrogradation promoters, retrogradation inhibitors, and those that inhibited retrogradation at low concentrations but promoted retrogradation at high concentrations (Figure 5). The retrogradation promoters were sweeteners that tended to increase retrogradation rates and amounts with increasing concentrations, and this group included xylitol, sorbitol, isomaltulose, isomalt, and raffinose (Figure 4). Trehalose was considered to be a retrogradation promoter since the Abs_∞_, and Abs_(∞-0)_ values of these gels increased with trehalose concentration; however, *k* values remained similar to the control (≈0.1 day^−1^). Isomalt and isomaltulose were two of the strongest retrogradation promoters and both have an α(1–6) glycosidic linkage, which allows for greater molecular extensibility and flexibility [58]. These two sweeteners also had the greatest effect on elevating the T_gel_ of starch at the 30% and 40% concentrations (Table 2). Retrogradation inhibitors were sweeteners that tended to decrease the amount of retrogradation compared with the control and included: ribose, xylose, allulose, sorbose, and tagatose. These sweeteners were among those that also had the least effect on elevating the T_gel_ (Table 2). It is important to note that retrogradation inhibitors did not always have low calculated *k* values. For example, when Abs_(∞-0)_ was ≈ 0.0, the calculated *k* values were artificially high (e.g., ribose and allulose in Figure 4). Sweeteners that acted as retrogradation inhibitors at low concentrations but as retrogradation promoters with increasing concentrations were: sucrose, glucose, mannose, galactose, fructose, maltose, and maltitol. Biliaderis and Prokopowich [38] previously noted that a sweetener could behave either as a retrogradation inhibitor or promoter based on concentration, documenting that fructose at low concentrations slowed retrogradation while high concentrations of fructose increased retrogradation. Lactose and mannitol were not classified due to solubility limitations. 

The information presented in Figure 5 could be useful for product developers when considering which sweeteners, at which concentrations, might create conditions favorable for the desirable amount of retrogradation in a given product. Sweeteners that inhibit retrogradation could be beneficial for use in products for which retrogradation is undesirable, e.g., to inhibit the staling of bread. In contrast, sweeteners that promote retrogradation could be used as a controlling formulation factor [59,60] to increase the amount of resistant starch type 3, which has documented health benefits. 

To better understand why different sweeteners affected retrogradation differently and at different concentrations, the effects of categorical sweetener solution properties (Table 1) on starch retrogradation in the wheat starch gels were investigated. The concentration of the sweetener solution and sweetener type (sugar alcohol or sugar) significantly affected the *k*, Abs_∞_, and Abs_(∞-0)_ values; the number of carbons was significant for Abs_(∞-0)_ but not *k* or Abs_∞_; and if the sweetener was a reducing sugar, it was not a significant factor (Table 3). Significant interactions were sweetener type with the number of carbons on Abs_∞_, and sweetener type with concentration on Abs_(∞-0)_ and *k* values. The gels that retrograded the most (i.e., highest Abs_∞_, and Abs_(∞-0)_ values) and the fastest (i.e., highest *k* values) were predominately made with sugar alcohols at high concentrations (40 and 50%), whereas the gels that retrograded the least and the slowest were made with sugars at low concentrations. Baek, Yoo and Lim [25] also reported sugar alcohols increased retrogradation of corn starch gels more than sugars. Therefore, a generalization is that a sugar solution at a low concentration would likely interfere with starch recrystallization, while a sugar alcohol solution, particularly at high concentrations, would likely promote starch crystallization.

**Figure 6:**
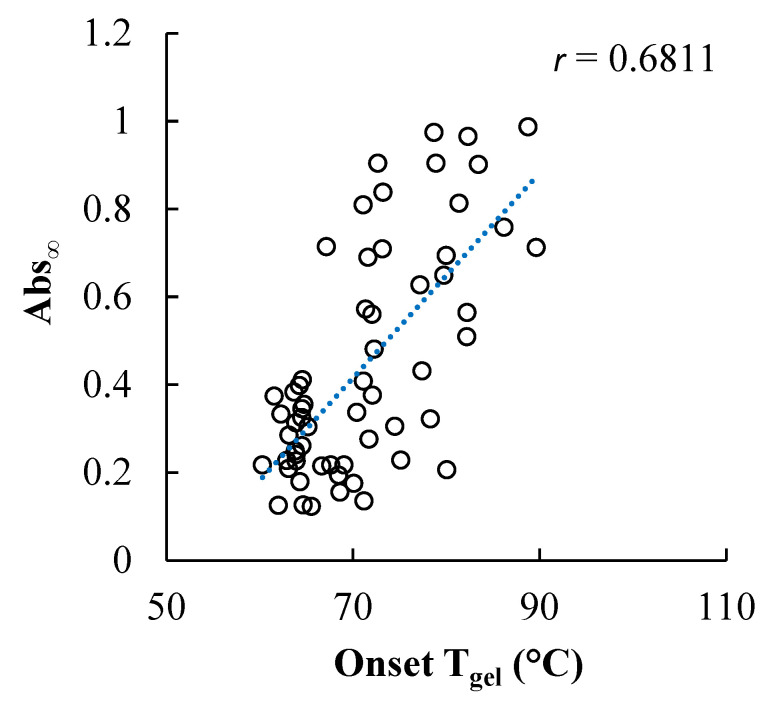
Relationship between the onset gelatinization temperature of wheat starch in solutions of the twenty sweetener solutions across a range of concentrations and a spectrophotometric measure of subsequent retrogradation of the starch in gels made using these same sweetener types and concentrations.

Moderate but significant correlations were found between the onset gelatinization temperature of wheat starch in the different sweetener solutions and retrogradation of the starch in gels made using these same sweetener types and concentrations (Figure 6, Table 4). The ϕ_w,eff_ was also moderately but significantly negatively correlated to Abs_∞,_ but to a lesser extent than T_gel_. Sweetener solutions that resulted in the highest onset T_gel_s also tended to result in the most retrogradation. Increasing the concentration of all of the sweeteners studied increased the T_gel_, although the extent of T_gel_ elevation varied across the different sweetener types attributed to structural differences between the sweeteners, including measures of their intermolecular hydrogen bonding ability and ϕ_w,eff_ [3,4]. It is important to note that more than the temperature differential between the onset T_gel_ and the preparation of the gels (99 °C) affected retrogradation, as reflected in the distribution of Abs_∞_ values at any given onset T_gel_ value (Figure 6). The amount and type of sweetener in solution affected the onset T_gel_ and would therefore have also likely altered the amount of residual short-range molecular order in the gelatinized starch as well as the molecular mobility of the glucan chains, factors shown to be influential in retrogradation [23].

To further separate the effects of different sweetener structural traits on retrogradation, linear correlations of the number of equatorial and exocyclic groups on a sweetener (Table 1) with starch gel retrogradation *k*, Abs_∞_, and Abs_(∞-0)_ values were investigated (Table 5). The number of equatorial and exocyclic hydroxyl groups on a sweetener in solution were negatively correlated with the *k* values of gels made with 10 and 20% sweetener concentrations but positively correlated with *k* values of gels made with 40 and 50% sweetener concentrations (Table 5 and Figure S1). In addition, the number of these hydroxyl groups was positively correlated with Abs_∞_ and Abs_(∞-0)_ values at 20 to 50% sweetener concentrations. The e-OH was more influential than the *N_OH,eff_*. The equatorial and exocyclic hydroxyl groups in a monosaccharide have been shown to be more reactive than axial hydroxyl groups [61]; thus, sweeteners with a greater amount of these more reactive hydroxyl groups are likely to form more intermolecular interactions. This suggests sweeteners with stereochemistries favorable for intermolecular interactions (i.e., H-bonding) may slow retrogradation rates at low concentrations but increase the amount and rate of starch retrogradation at high concentrations. Sweetener stereochemistry and the orientation of the hydroxyl groups have been associated with many phenomena that likely influence retrogradation including: how a sweetener fits in the structure of water [49], the extent to which a sweetener increases the gelatinization temperature of starch [3], the amount of unfreezable water and changes of solution specific heat [62,63], the dynamic hydration number of the sweetener [64], the intrinsic viscosity of the sweetener [45], and the diffusion coefficient of the sweetener [65]. The stereochemistry of sweeteners has also been correlated with their effects on starch retrogradation rates. Muira, Nishimura, and Katsuta [33] and Katsuta, Nishimura, and Miura [31] reported negative correlations between the retrogradation rate constants (*k*) in a first-order kinetic equation of 6% sweetener and 30% rice starch gels and the number of equatorial hydroxyl groups in the sweeteners. This is in agreement with the retrogradation behaviors of wheat starch gels made with 10 and 20% sweetener solutions because there were also negative correlations with the retrogradation rate constants (*k*) and the number of equatorial and exocyclic hydroxyl groups in a sweetener (Figure 6). 

Sweetener molar volumes (Table 1) were also significantly correlated with retrogradation *k* values of gels made with 20 and 30% sweetener solutions and the Abs_∞_ and Abs_(∞-0)_ values of gels made with 20, 30, and 40% sweetener solutions (Table 5 and Figure S1). However, the slopes of the significant correlations (Table 5 and Figure S1) were ≈10 to 100x less than the slopes of correlations with the number of equatorial and exocyclic hydroxyl groups (Table 5 and Figure S1), indicating the effect of the sweetener molar volume on the retrogradation behavior may not be as great as the effect of the sweetener stereochemistry. Despite lower impacts, sweeteners with larger molar volumes were more likely to increase the amount of retrogradation (Abs_∞_ and Abs_(∞-0)_) without greatly affecting retrogradation rates (*k*). The molar volume is the space the solute takes up in solution [44], so conceptually, sweeteners with a larger molar volume could span further distances to form hydrogen bond bridges between starch chains, which could initiate retrogradation. 

The *k*, Abs_∞_, and Abs_(∞-0)_ values of retrogradation were also compared with the dry glass transition temperatures (T_g_) of sweeteners (Table 1). The slopes of these correlations were ≈0 and few correlations were statistically significant (Table 5, Figure S1). It was therefore concluded that the dry T_g_ of a sweetener did affect the retrogradation of these 10%*w*/*v* gels. Slade et al. [66] proposed sweeteners delay retrogradation because they function as antiplasticizers, restricting starch chain mobility and thereby slowing or preventing recrystallization. The T_g_s of the 10%*w*/*v* starch gels in this study were likely not greatly affected by the presence of sweeteners because the water content was high and the T_g_ values of the gels were much lower than the storage temperature (4 °C). For example, the T_g_ of the wheat starch gel made with a 50% sucrose solution was estimated to be ≈−63 °C (210 K) using the Fox equation (1/T_g*,i*_ = Σ*_i_ w_i_*/T_g,*i*_ where *w_i_* is the mass fraction of the component *i*, and T_g*,i*_ is the dry T_g_ of component *i* [67]; T_g_ of starch was 416 K [68], T_g_ of sucrose was 333 K (Table 1), and T_g_ of water was 144 K [69]). Therefore, the T_g_ values of the gels were well below the storage condition and T_g_ differences from the varying sweeteners and concentrations did not affect the retrogradation behaviors.

### 3.3. Retrogradation Promoting and Inhibiting Traits

Different sweeteners promoted or inhibited retrogradation across all concentrations, while some sweeteners had a concentration-dependent effect on retrogradation (Figure 5). For a sweetener to impact retrogradation, it will affect one or more the reported stages of retrogradation: (1) double helix formation without true crystallinity, (2) an induction time before crystal growth, (3) primary crystallization wherein measurable crystalline regions form, and (4) crystal propagation and perfecting [70]. The influence of the sweetener on the T_gel_ of the starch and retention of residual short-range molecular order in the gelatinized starch [23] must also be considered. All sweeteners increased the T_gel_ relative to the water control, yet some sweeteners delayed retrogradation compared with the control. These sweeteners that delayed retrogradation likely functioned as starch crystallization impurities and contained structures less favorable for intermolecular interactions. The sweeteners that inhibited retrogradation (ribose, xylose, tagatose, allulose, and sorbose) tended to have the least effect on elevating the T_gel_ (Table 2), another indicator that their interactions with starch were less favorable than other sweeteners in the study. In gels with higher sweetener concentrations and/or with sweeteners that have structures more favorable for intermolecular interactions, the sweeteners functioned as bridges between starch chains [24] and competed for water molecules [38], both forces promoting starch–starch interactions. More retrogradation occurred in the presence of sweetener types (e.g., isomalt, isomaltulose, and sorbitol) and concentrations that tended to also elevate T_gel_ the most, indicative of the favorable intermolecular interactions and stereochemistries, such as the sugar alcohols, which have flexible open structures [71], and the sweeteners with α(1–6) linkages (isomalt and isomaltulose) [58].

The concentration-dependent influence of some of the sweeteners on inhibiting retrogradation at lower concentrations then promoting retrogradation at higher concentrations underscores that more than one factor affects starch retrogradation in the presence of the same sweetener. The retrogradation behaviors (*k*, Abs_∞_, and Abs_(∞-0)_) of starch gels made with sucrose, glucose, mannose, galactose, fructose, maltose, and maltitol were not linear with concentration but instead were primarily “U”-shaped (Figure 2 and Figure S1). Increasing sweetener concentration decreases water content and increases T_gel_ (and in turn T-T_gel_ and associated residual short-range molecular order). Huang, Chao, Yu, Copeland, and Wang [23] reported that retrogradation of wheat starch (in the absence of sugars) is dependent on a balance of the molecular mobility of glucan chains and the amount of residual short-range molecular order and that there is an optimal amount of short-range molecular order determined by the heating temperature and water content that will favor retrogradation. In that study, retrogradation initially increased as sample treatment decreased the residual short-range molecular order but further decreases in the molecular order decreased retrogradation. The addition of sweeteners to the system would have altered both the glucan mobility and amount of residual starch structure, as well as the structure and mobility of water. It is interesting to note that the sweeteners with the concentration-dependent inhibition and then promotion effects on retrogradation relative to the control were the sweeteners that tended to have intermediate effects on the elevation of the starch T_gel_. Higher sweetener concentrations increased T_gel_ and would have retained more residual starch structure, which likely served as nucleation sites and promoted retrogradation. At the lower sweetener concentrations, despite having higher T_gel_s and, therefore, more residual short-range order than the water control, the number of equatorial and exocyclic hydroxyl groups in the sweeteners was negatively correlated with retrogradation. It could be that the favorable intermolecular hydrogen bonding between these sweeteners and starch resulted in interference with crystal growth in these conditions, but not in systems containing higher amounts of these sweeteners and more residual short-range order.

## 4. Conclusions

The effects of twenty sweeteners across a range of concentrations on the retrogradation of wheat starch gels fit into three general categories: retrogradation promoters, retrogradation inhibitors, and retrogradation inhibitors at low concentrations and promoters at high concentrations. Gels tended to retrograde more and faster with higher sweetener concentrations (≥40%) and with sweeteners with molecular configurations favorable for intermolecular interactions, such as more equatorial and exocyclic hydroxyl groups, α(1,6) linkages, larger molar volumes, and sugar alcohols. The sweeteners that promoted retrogradation also tended to elevate the T_gel_ to a greater extent, an indicator of favorable and stabilizing intermolecular interactions between the sweetener and starch glucans. Retrogradation tended to be inhibited in the presence of low sweetener concentrations and sweeteners with configurations less favorable for intermolecular interactions. It is important to consider how different sweeteners at different concentrations affect starch retrogradation, given the wide landscape of sweetener type and concentration effects on retrogradation rates and amounts. While useful for improving the understanding of how different sweeteners influence starch retrogradation, these findings can also help guide food product developers in selecting sweeteners for starchy products for which retrogradation is either desirable or undesirable. 

## Figures and Tables

**Table 1:** Sugar and sugar alcohol properties.

Sweetener	# of Carbons	Type	Reducing Sugar	Tg (°C)	Exocyclic + Equatorial OH (Table S1)	Molar Volume (cm^3^/mole)
Allulose	6	Sugar (Ketose)	Yes	0.5	2.7	
Fructose	6	Sugar (Ketose)	Yes	15.2 [3]	2.7	110.4 [44]
Galactose	6	Sugar (Aldose)	Yes	31.9 [3]	3.6	111.9 [44]
Glucose	6	Sugar (Aldose)	Yes	38.3 [3]	4.6	112.2 [44]
Isomalt	12	Sugar Alcohol	No	58.7 [3]	9.0	
Isomaltulose	12	Sugar	Yes	60.6 [3]	5.2	219.5 [45]
Lactose	12	Sugar	Yes	101.0 [46]	6.6	207.6 [44]
Maltitol	12	Sugar Alcohol	No	46.4 [3]	9.0	215.4 [47]
Maltose	12	Sugar	Yes	49.0 [3]	7.4	208.8 [44]
Mannitol	6	Sugar Alcohol	No	10.7 [48]	6.0	119.4 [49]
Mannose	6	Sugar (Aldose)	Yes	35.9 [3]	3.3	111.7 [44]
Raffinose	18	Sugar	No	103.2 [50]	8.0	303.2 [44]
Ribose	5	Sugar (Aldose)	Yes	−11.6 [3]	2.4	95.3 [44]
Sorbitol	6	Sugar Alcohol	No	−1.6 [3]	6.0	119.9 [49]
Sorbose	6	Sugar (Ketose)	Yes	19.0 [46]	4.0	110.6 [44]
Sucrose	12	Sugar	No	59.4 [3]	6.0	210.2 [44]
Tagatose	6	Sugar (Ketose)	Yes	14.1 [3]	3.1	108.9 [51]
Trehalose	12	Sugar	No	117.5 [3]	8.0	206.9 [44]
Xylitol	5	Sugar Alcohol	No	−23.8 [3]	5.0	102.4 [49]
Xylose	5	Sugar (Aldose)	Yes	11.0 [3]	3.6	94.8 [44]

#: Number of carbons.

**Table 2:** The onset gelatinization temperature (T_gel_) of wheat starch in the presence of different concentrations of sweetener solutions (10% to 50%).

	Onset T_gel_ (°C)			
Sweetener	10%	30%	40%	50%
Ribose	60.28 ± 0.25	62.00 ± 0.10	64.67 ± 0.22	68.62 ± 0.19
Xylose	61.55 ± 0.49	63.91 ± 0.10	67.63 ± 0.05	72.07 ± 0.12
Xylitol	62.90 ± 0.09	67.15 ± 0.30	72.66 ± 0.27	78.69 ± 0.34
Tagatose	63.13 ± 0.12	66.65 ± 0.17	71.71 ± 0.18	78.30 ± 0.20
Mannose	63.90 ± 0.11	68.45 ± 0.24	74.48 ± 0.12	82.20 ± 0.07
Fructose	63.87 ± 0.13	69.03 ± 0.09	75.12 ± 0.19	82.22 ± 0.10
Allulose	62.28 ± 0.03	65.54 ± 0.08	71.19 ± 0.08	80.05 ± 0.38
Galactose	64.53 ± 0.17	71.12 ± 0.42	N/A	N/A
Glucose	64.54 ± 0.07	70.41 ± 0.19	77.17 ± 0.50	86.19 ± 0.64
Sorbitol	64.76 ± 0.11	71.62 ± 0.67	78.90 ± 0.38	88.75 ± 0.02
Mannitol	65.18 ± 0.11	N/A	N/A	N/A
Maltose	63.10 ± 0.61	70.10 ± 0.13	77.39 ± 0.37	N/A
Trehalose	64.51 ± 0.24	71.36 ± 0.17	79.73 ± 0.17	N/A
Sucrose	64.31 ± 0.12	72.28 ± 0.16	79.98 ± 0.06	89.64 ± 0.12
Maltitol	63.82 ± 0.19	72.04 ± 0.21	81.36 ± 0.22	N/A
Isomalt	64.26 ± 0.47	73.21 ± 0.53	82.33 ± 0.49	N/A
Isomaltulose	63.67 ± 0.32	73.15 ± 0.30	83.45 ± 0.48	N/A
Raffinose	64.57 ± 0.08	71.10 ± 0.69	N/A	N/A

**Table 3:** *p*-values of retrogradation properties and significant *p*-values are bolded (α = 0.05).

	*p* Value		
Source	*k* (day^−1^)	Abs_∞_	Abs_(∞-0)_
# of Carbons	0.455	0.072	**0.027**
Sugar vs. Sugar Alcohol	**0.002**	**0.001**	**<0.001**
Reducing Sugar	0.075	0.711	0.125
Concentration	**<0.001**	**<0.001**	**<0.001**

#: Number of carbons.

**Table 4:** Pearson correlation coefficient (*r*) of linear correlations of wheat starch gel retrogradation properties (*k*, Abs_∞_, Abs_(∞-0)_) with sweetener solution properties (*N_OH,eff_*, and ϕ_w,eff_ calculated as shown in Table S2) and the onset gelatinization temperature of starch in the sweetener solutions.

	Correlation Coefficient (*r*)		
Sweetener Solution Parameter	*k* (day^−1^)	Abs_∞_	Abs_(∞-0)_
Onset T_gel_	0.1700	0.6811 ***	0.6077 ***
*N_OH,eff_*	−0.1263	−0.1882	−0.1593
ϕ_w,eff_	−0.1768	−0.5009 ***	−0.4062 **

** *p*-value < 0.01, *** *p*-value < 0.001.

**Table 5:** Pearson correlation coefficient (*r*), slope, and *p*-values of linear correlations of starch gel retrogradation properties (*k*, Abs_∞_, Abs_(∞-0)_) in respect to sweetener factors (number of equatorial + exocyclic hydroxyl groups (e-OH), dry T_g_, molar volume, *N_OH,eff_*, and ϕ_w,eff_) with correlations grouped by sweetener concentrations. Correlations with *p*-values < 0.10 are indicated by * and *p*-values < 0.05 are indicated by **. Sample size of correlations varied because of solubility limitations and missing values in Table 1.

Sweetener Concentration	Sweetener Factor			*k*				Abs_∞_				Abs_(∞-0)_		
				*r*	Slope	*p*-Value		*r*	Slope	*p*-Value		*r*	Slope	*p*-Value
10	e-OH			0.562	−0.013	0.012 **		0.166	0.005	0.484		0.197	0.005	0.405
20	e-OH			0.556	−0.012	0.017 **		0.503	0.031	0.028 **		0.498	0.022	0.030 **
30	e-OH			0.407	0.015	0.116		0.715	0.080	0.001 **		0.667	0.046	0.002 **
40	e-OH			0.525	0.034	0.045 **		0.746	0.103	0.001 **		0.703	0.054	0.002 **
50	e-OH			0.548	0.058	0.097 *		0.711	0.097	0.010 **		0.595	0.049	0.041 **
10	Dry T_g_			0.492	−0.001	0.037 **		0.238	0.000	0.326		0.234	0.000	0.333
20	Dry T_g_			0.468	−0.001	0.057 *		0.342	0.001	0.164		0.335	0.001	0.174
30	Dry T_g_			0.352	0.001	0.179		0.434	0.003	0.081 *		0.361	0.002	0.154
40	Dry T_g_			0.118	0.000	0.686		0.339	0.003	0.215		0.293	0.001	0.288
50	Dry T_g_			0.366	−0.003	0.294		0.173	0.002	0.610		0.171	0.001	0.613
10	Molar Volume			0.405	0.000	0.107		0.164	0.000	0.516		0.258	0.000	0.302
20	Molar Volume			0.470	0.000	0.067 *		0.471	0.001	0.056 *		0.541	0.001	0.025 **
30	Molar Volume			0.570	0.001	0.027 **		0.579	0.002	0.019 **		0.522	0.001	0.038 **
40	Molar Volume			0.370	0.001	0.213		0.519	0.003	0.057 *		0.504	0.002	0.066 *
50	Molar Volume			0.131	0.001	0.737		0.349	0.002	0.323		0.279	0.001	0.435
10	*N_OH,eff_*			0.416	1.296	0.085 *		0.012	0.000	0.961		0.149	−0.003	0.541
20	*N_OH,eff_*			0.198	0.120	0.430		0.325	−0.565	0.175		0.466	−0.585	0.050 *
30	*N_OH,eff_*			0.460	−0.071	0.071 *		0.398	0.079	0.112		0.393	−0.537	0.106
40	*N_OH,eff_*			0.589	−0.530	0.025 **		0.506	−1.002	0.053 *		0.560	−0.615	0.029 **
50	*N_OH,eff_*			0.322	−0.340	0.361		0.358	1.214	0.253		0.299	0.990	0.346
10	ϕ_w,eff_			0.114	1.296	0.663		0.128	2.106	0.614		0.066	−0.842	0.795
20	ϕ_w,eff_			0.275	0.000	0.303		0.363	−5.817	0.152		0.471	−5.352	0.056 *
30	ϕ_w,eff_			0.244	−1.439	0.382		0.407	0.002	0.118		0.422	−5.101	0.103
40	ϕ_w,eff_			0.111	0.001	0.717		0.088	−1.026	0.765		0.094	−0.609	0.750
50	ϕ_w,eff_			0.614	−5.705	0.078*		0.607	2.160	0.063 *		0.500	1.632	0.141

## Data Availability

Data is contained within the article or supplementary material.

## Supplementary Materials

The following supporting information can be downloaded at https://www.mdpi.com/article/10.3390/foods11193008/s1, Table S1. Thermal properties of wheat starch slurries (gelatinization) and gels (retrogradation) made with 10 to 50% sucrose solutions. Enthalpies were not adjusted to dry starch content. Capital letters indicate significant differences. Table S2. Anomeric and tautomeric forms of sweeteners in solution and the number of equatorial and exocyclic hydroxyl groups. Table S3. Calculated sweetener solution *N_OH,eff_*, and ϕ_w,eff_ values. Figure S1. Plots of 620 nm absorbances after 28 days (Abs_∞_) with one standard deviation, changes in 620 nm absorbances from day 0 to day 28 (Abs_(∞-0)_) with one standard deviation, and the calculated Avrami rate constants (*k*) with 95% confidence intervals that were grouped by sweetener concentrations within sweeteners.
