# Supplementary material for: Variable Effects of Twenty Sugars and Sugar Alcohols on the Retrogradation of Wheat Starch Gels
Source: Foods. 2022 Sep 27;11(19):3008. doi: 10.3390/foods11193008 (PMC9563978; doi:10.3390/foods11193008)
Supplement: Supplementary file 1 [file foods-11-03008-s001.zip › foods-1901575-supplementary.pdf]

**Table S1.** Thermal properties of wheat starch slurries (gelatinization) and gels (retrogradation) made with 10 to 50% sucrose solutions. Enthalpies were not adjusted to dry starch content. Capital letters indicate significant differences.

| | **Gelatinization** | | | | | | | | | | | |
|---|---|---|---|---|---|---|---|---|---|---|---|---|
| | Onset (°C) | | | | Peak (°C) | | | | Enthalpy (J/g) | | | |
| | Avg. | | SD | Stat. | Avg. | | SD | Stat. | Avg. | | SD | Stat. |
| **Control** | 60.60 | ± | 0.30 | F | 64.70 | ± | 0.15 | F | 0.82 | ± | 0.09 | B |
| **10% Sucrose** | 63.40 | ± | 0.01 | E | 67.52 | ± | 0.01 | E | 0.69 | ± | 0.04 | B |
| **20% Sucrose** | 66.66 | ± | 0.18 | D | 71.02 | ± | 0.16 | D | 0.68 | ± | 0.09 | B |
| **30% Sucrose** | 71.42 | ± | 0.07 | C | 75.74 | ± | 0.10 | C | 0.62 | ± | 0.02 | B |
| **40% Sucrose** | 76.66 | ± | 0.84 | B | 81.73 | ± | 0.11 | B | 0.91 | ± | 0.30 | AB |
| **50% Sucrose** | 82.75 | ± | 1.35 | A | 89.57 | ± | 0.25 | A | 1.26 | ± | 0.22 | A |
| | **Retrogradation Day 7** | | | | | | | | | | | |
| | Onset (°C) | | | | Peak (°C) | | | | Enthalpy (J/g) | | | |
| | Avg. | | SD | Stat. | Avg. | | SD | Stat. | Avg. | | SD | Stat. |
| **Control** | 48.85 | ± | 2.56 | A | 55.07 | ± | 0.75 | C | 0.04 | ± | 0.02 | D |
| **10% Sucrose** | 49.49 | ± | 2.46 | A | 55.59 | ± | 0.37 | BC | 0.03 | ± | 0.00 | D |
| **20% Sucrose** | 46.45 | ± | 2.18 | A | 53.68 | ± | 0.78 | C | 0.11 | ± | 0.03 | CD |
| **30% Sucrose** | 46.15 | ± | 2.03 | A | 55.69 | ± | 2.01 | BC | 0.14 | ± | 0.02 | BC |
| **40% Sucrose** | 47.58 | ± | 1.15 | A | 58.49 | ± | 1.03 | AB | 0.23 | ± | 0.07 | AB |
| **50% Sucrose** | 47.92 | ± | 0.62 | A | 60.32 | ± | 1.18 | AB | 0.27 | ± | 0.02 | A |
| | **Retrogradation Day 14** | | | | | | | | | | | |
| | Onset (°C) | | | | Peak (°C) | | | | Enthalpy (J/g) | | | |
| | Avg. | | SD | Stat. | Avg. | | SD | Stat. | Avg. | | SD | Stat. |
| **Control** | 49.40 | ± | 4.00 | A | 55.88 | ± | 0.74 | BC | 0.03 | ± | 0.02 | E |
| **10% Sucrose** | 44.07 | ± | 0.49 | AB | 53.19 | ± | 1.81 | C | 0.06 | ± | 0.01 | DE |
| **20% Sucrose** | 42.88 | ± | 0.54 | B | 52.65 | ± | 1.13 | C | 0.11 | ± | 0.02 | CD |
| **30% Sucrose** | 45.51 | ± | 0.95 | AB | 56.51 | ± | 1.00 | B | 0.14 | ± | 0.01 | BC |
| **40% Sucrose** | 47.64 | ± | 3.01 | AB | 58.70 | ± | 0.77 | AB | 0.19 | ± | 0.03 | B |
| **50% Sucrose** | 47.61 | ± | 0.60 | AB | 60.96 | ± | 0.38 | A | 0.27 | ± | 0.03 | A |

**Table S2.** Anomeric and tautomeric forms of sweeteners in solution and the number of equatorial and exocyclic hydroxyl groups.

| | Mutarotation Distribution | | | | | | # of Equatorial OH Groups and Exocyclic OH Groups | | | | | Calculated equ. and exo-OH | Reported e-OH | Reference |
|---|---|---|---|---|---|---|---|---|---|---|---|---|---|---|
| | Pyranose | | Furanose | | | | Pyranose | | Furanose | | | | | |
| Sweetener | α | β | α | β | Open | Reference | α | β | α | β | Open | | | |
| Ribose | 21.5 | 58.5 | 6.5 | 13.5 | 0.05 | [1] | 2 | 3 | 1 | 1 | 4 | 2.4 | 2.1 | [6] |
| Xylose | 36.5 | 63 | 0.25 | 0.25 | 0.02 | [1] | 3 | 4 | 1 | 1 | 4 | 3.6 | 3.5 | [6] |
| Xylitol | NA | NA | NA | NA | 100 | | NA | NA | NA | NA | 5 | 5.0 | | |
| Glucose | 38 | 62 | 0.0 | 0.14 | 0.002 | [1] | 4 | 5 | 2 | 2 | 5 | 4.6 | 4.6 | [6] |
| Galactose | 30 | 64 | 2.5 | 3.5 | 0.02 | [1] | 3 | 4 | 2 | 2 | 5 | 3.6 | 3.6 | [6] |
| Mannose | 65.5 | 34.5 | 0.6 | 0.3 | 0.005 | [1] | 3 | 4 | 2 | 2 | 5 | 3.4 | 3.3 | [6] |
| Fructose | 2 | 70 | 5 | 23 | 0.7 | [1] | 2 | 3 | 2 | 2 | 5 | 2.7 | 3.0 | [6] |
| Tagatose | 79 | 16 | 1 | 4 | 0.6 | [1] | 3 | 4 | 2 | 2 | 5 | 3.1 | | |
| L-Sorbose | 98 | 0 | 2 | 0 | 0.2 | [2] | 4 | 5 | 2 | 2 | 5 | 4.0 | 3.9 | [7] |
| Sorbitol | NA | NA | NA | NA | 100 | | NA | NA | NA | NA | 6 | 6.0 | | |
| Mannitol | NA | NA | NA | NA | 100 | | NA | NA | NA | NA | 6 | 6.0 | | |
| Maltose | 62 | 38 | 0 | 0 | 0 | [3] | 7 | 8 | 6 | 6 | 10 | 7.4 | 7.2 | [6] |
| Trehalose | 100 | 0 | 0 | 0 | 0 | | 8 | NA | NA | NA | NA | 8.0 | 8.0 | [8] |
| Sucrose | 0 | 0 | 0 | 100 | 0 | | NA | NA | NA | 6 | NA | 6.0 | 6.3 | [6] |
| Isomaltulose | 0 | 0 | 25 | 69 | 6 | [4] | 5 | 6 | 5 | 5 | 9 | 5.2 | | |
| Lactose | 37.3 | 62.7 | 0 | 0 | 0 | [5] | 6 | 7 | 5 | 5 | 9 | 6.6 | 6.7 | [9] |
| Isomalt | NA | NA | NA | NA | 100 | | NA | NA | NA | NA | 9 | 9.0 | | |
| Raffinose | 0 | 0 | 0 | 100 | 0 | | NA | NA | NA | NA | NA | 8.0 | 8.3 | [6] |

**Table S3.** Calculated sweetener solution $N_{OH,eff}$ , and $\phi_{w,eff}$ values.

| Sweetener | Concentration | $N_{OH,eff}$ | $\phi_{w,eff}$ |
|---|---|---|---|
| Control | 0 | 2.000 | 1.000 |
| Allulose | 10 | 2.015 | |
| Fructose | 10 | 2.018 | 0.955 |
| Galactose | 10 | 2.021 | 0.956 |
| Glucose | 10 | 10.034 | 0.957 |
| Isomalt | 10 | 10.016 | |
| Isomaltulose | 10 | 10.017 | 0.946 |
| Lactose | 10 | 10.184 | 0.955 |
| Maltitol | 10 | 10.114 | 0.947 |
| Maltose | 10 | 10.117 | 0.949 |
| Mannitol | 10 | 10.182 | 0.950 |
| Mannose | 10 | 10.214 | 0.957 |
| Raffinose | 10 | 10.124 | 0.950 |
| Ribose | 10 | 10.199 | 0.954 |
| Sorbitol | 10 | 10.170 | 0.949 |
| Sorbose | 10 | 10.193 | 0.955 |
| Sucrose | 10 | 10.127 | 0.949 |
| Tagatose | 10 | 10.188 | 0.956 |
| Trehalose | 10 | 10.222 | 0.959 |
| Xylitol | 10 | 10.185 | 0.949 |
| Xylose | 10 | 10.223 | 0.956 |
| Allulose | 20 | 9.250 | |
| Fructose | 20 | 9.279 | 0.906 |
| Galactose | 20 | 9.318 | 0.908 |
| Glucose | 20 | 9.335 | 0.910 |

| | | | |
|---|---|---|---|
| Isomalt | 20 | 9.152 | |
| Isomaltulose | 20 | 9.157 | 0.889 |
| Lactose | 20 | 9.268 | 0.906 |
| Maltitol | 20 | 9.130 | 0.889 |
| Maltose | 20 | 9.136 | 0.893 |
| Mannose | 20 | 9.329 | 0.910 |
| Raffinose | 20 | 9.149 | 0.896 |
| Ribose | 20 | 9.298 | 0.904 |
| Sorbitol | 20 | 9.242 | 0.894 |
| Sorbose | 20 | 9.287 | 0.907 |
| Sucrose | 20 | 9.155 | 0.894 |
| Tagatose | 20 | 9.276 | 0.907 |
| Trehalose | 20 | 9.345 | 0.914 |
| Xylitol | 20 | 9.270 | 0.894 |
| Xylose | 20 | 9.348 | 0.909 |
| Allulose | 30 | 8.325 | |
| Fructose | 30 | 8.369 | 0.853 |
| Galactose | 30 | 8.428 | 0.857 |
| Glucose | 30 | 8.453 | 0.859 |
| Isomalt | 30 | 8.178 | |
| Isomaltulose | 30 | 8.353 | 0.853 |
| Maltitol | 30 | 8.146 | 0.827 |
| Maltose | 30 | 8.154 | 0.832 |
| Mannose | 30 | 8.443 | 0.859 |
| Raffinose | 30 | 8.174 | 0.837 |
| Ribose | 30 | 8.398 | 0.850 |
| Sorbitol | 30 | 8.313 | 0.834 |

| | | | |
|---|---|---|---|
| Sorbose | 30 | 8.381 | 0.854 |
| Sucrose | 30 | 8.183 | 0.834 |
| Tagatose | 30 | 8.365 | 0.855 |
| Trehalose | 30 | 8.467 | 0.866 |
| Xylitol | 30 | 8.356 | 0.835 |
| Xylose | 30 | 8.473 | 0.858 |
| Allulose | 40 | 7.400 | |
| Fructose | 40 | 7.459 | 0.795 |
| Glucose | 40 | 7.572 | 0.803 |
| Isomalt | 40 | 7.205 | |
| Isomaltulose | 40 | 7.215 | 0.856 |
| Maltitol | 40 | 7.161 | 0.759 |
| Maltose | 40 | 7.173 | 0.766 |
| Mannose | 40 | 7.558 | 0.803 |
| Ribose | 40 | 7.498 | 0.791 |
| Sorbitol | 40 | 7.385 | 0.771 |
| Sorbose | 40 | 7.475 | 0.796 |
| Sucrose | 40 | 7.211 | 0.768 |
| Tagatose | 40 | 7.454 | 0.798 |
| Trehalose | 40 | 7.590 | 0.812 |
| Xylitol | 40 | 7.442 | 0.771 |
| Xylose | 40 | 7.597 | 0.803 |
| Allulose | 50 | 6.476 | |
| Fructose | 50 | 6.549 | 0.732 |
| Glucose | 50 | 6.690 | 0.743 |
| Maltitol | 50 | 6.177 | 0.685 |
| Maltose | 50 | 6.192 | 0.693 |

| | | | |
|---|---|---|---|
| Mannose | 50 | 6.673 | 0.742 |
| Ribose | 50 | 6.598 | 0.727 |
| Sorbitol | 50 | 6.456 | 0.701 |
| Sucrose | 50 | 6.238 | 0.696 |
| Tagatose | 50 | 6.543 | 0.735 |
| Xylitol | 50 | 6.528 | 0.703 |
| Xylose | 50 | 6.722 | 0.742 |

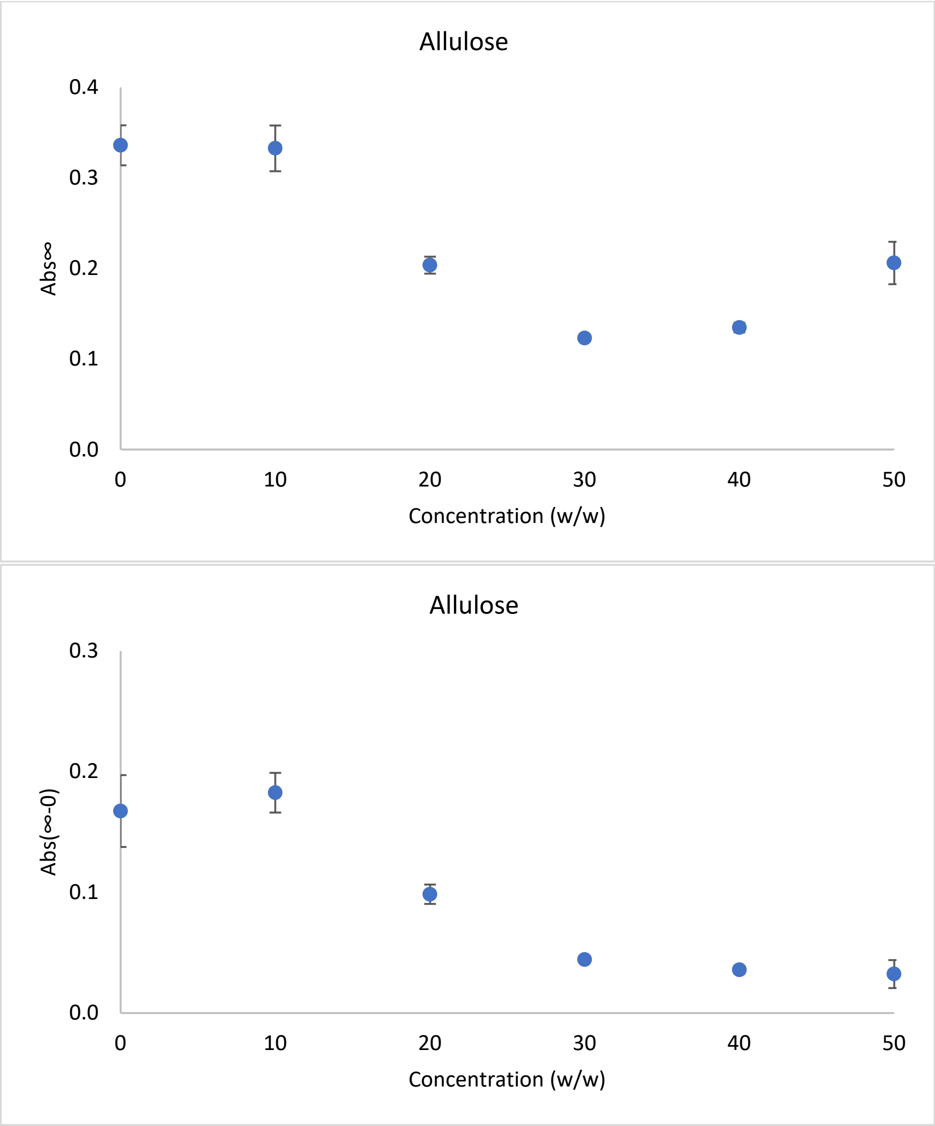
Allulose
0.4
0.3
0.2
0.1
0.0
Abs∞
0
10
20
30
40
50
Concentration (w/w)
Allulose
0.3
0.2
0.1
0.0
Abs(∞-0)
0
10
20
30
40
50
Concentration (w/w)

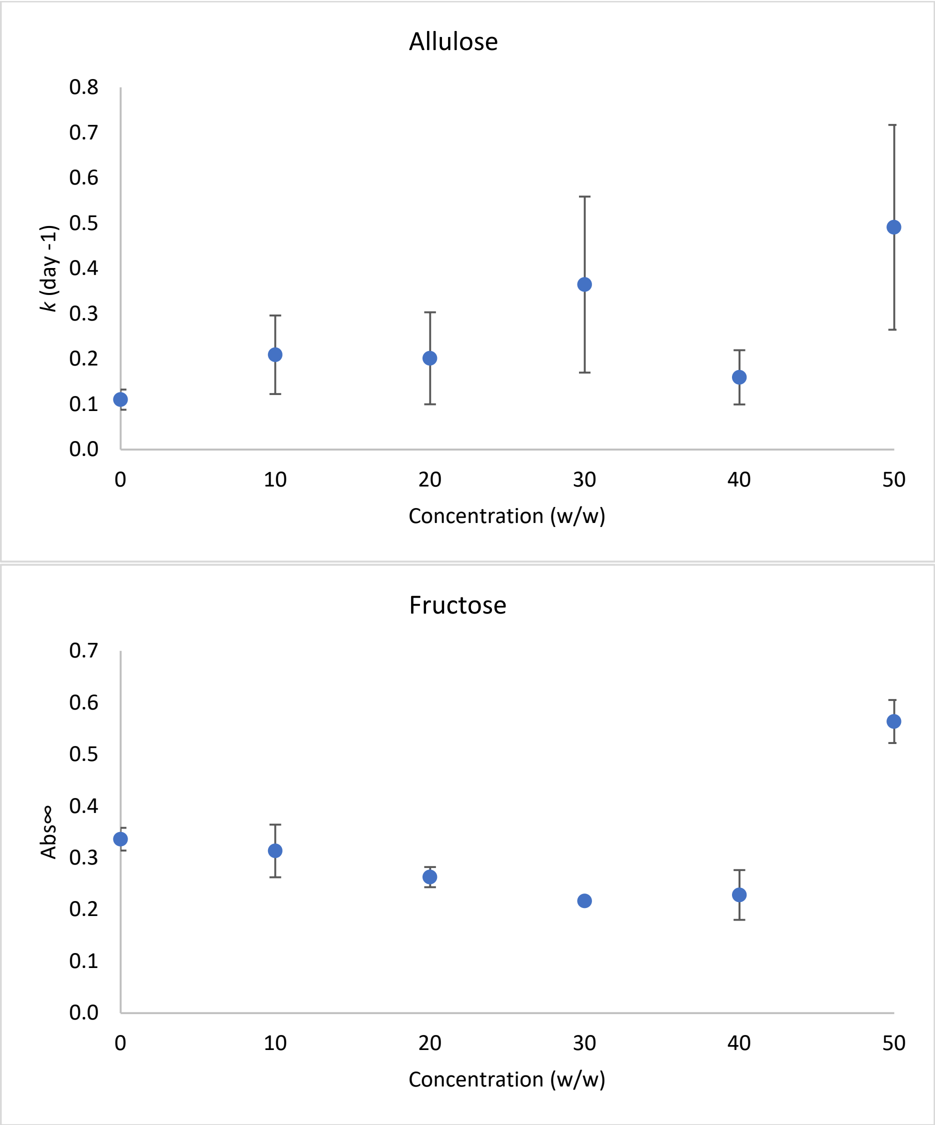
Allulose
k (day -1)
0.0
0.1
0.2
0.3
0.4
0.5
0.6
0.7
0.8
0
10
20
30
40
50
Concentration (w/w)
Fructose
Abs∞
0.0
0.1
0.2
0.3
0.4
0.5
0.6
0.7
0
10
20
30
40
50
Concentration (w/w)

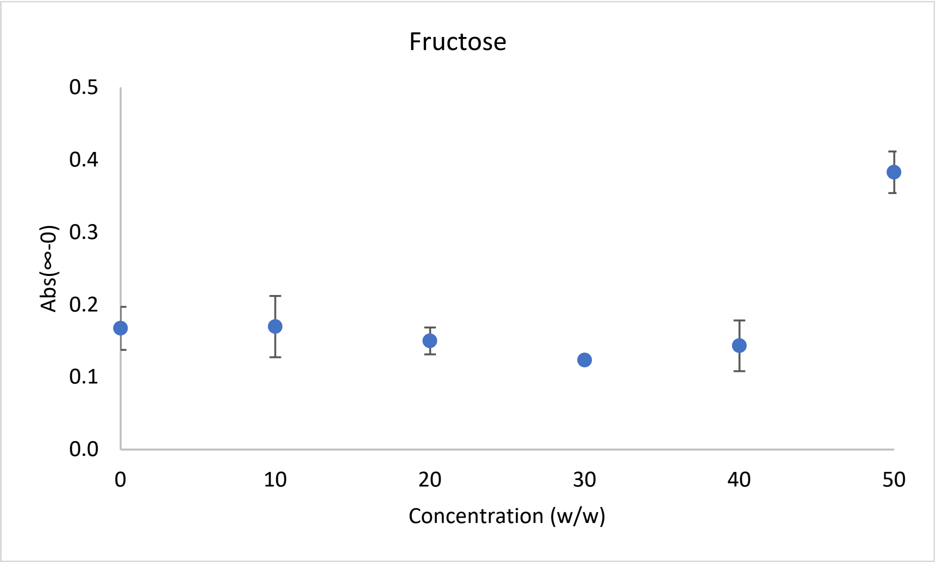
Fructose
0.5
0.4
0.3
0.2
0.1
0.0
Abs(∞-0)
0
10
20
30
40
50
Concentration (w/w)

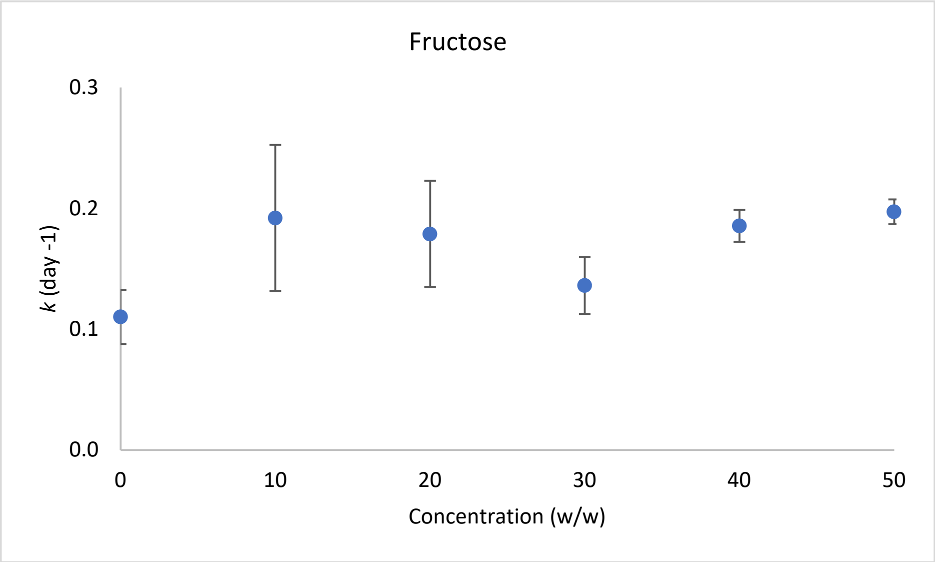
Fructose
0.3
0.2
0.1
0.0
k (day -1)
0
10
20
30
40
50
Concentration (w/w)

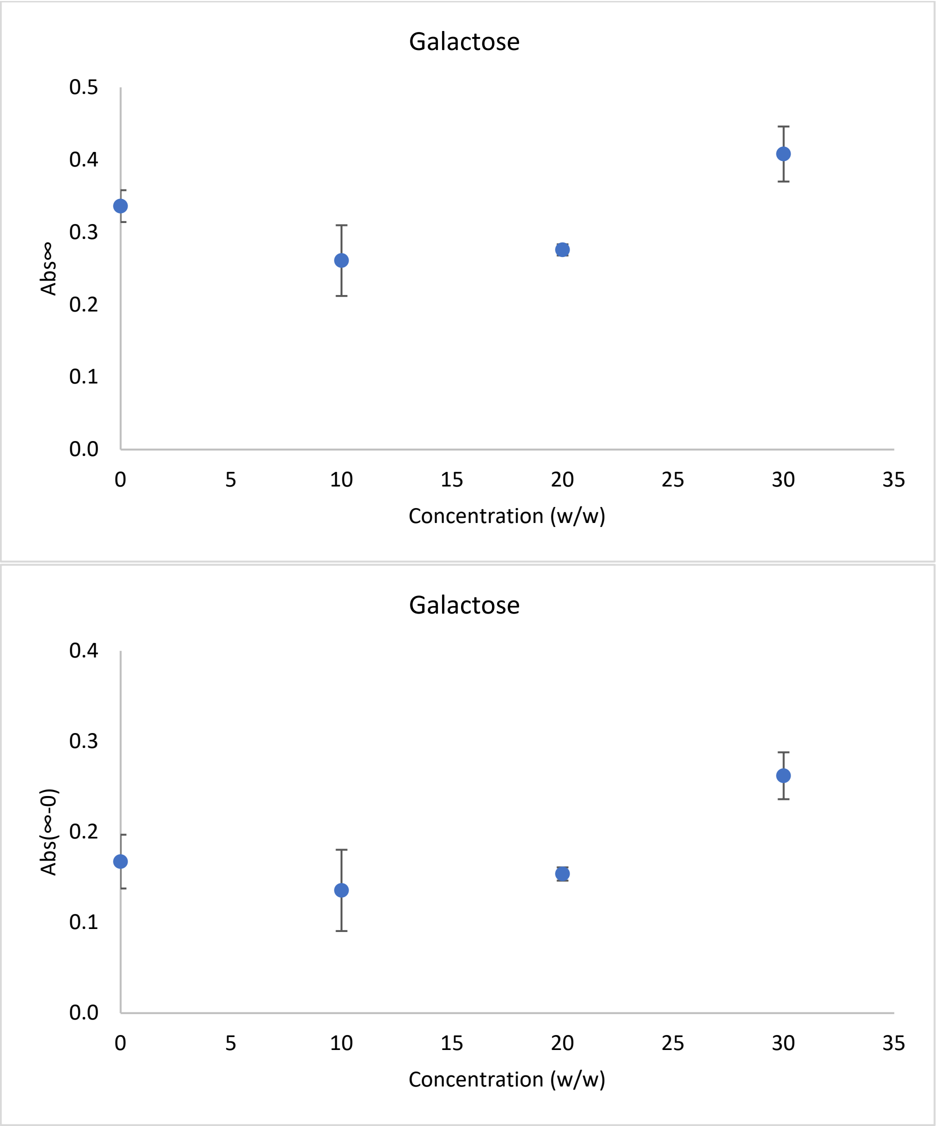
Galactose
0.5
0.4
0.3
0.2
0.1
0.0
Abs∞
0
5
10
15
20
25
30
35
Concentration (w/w)
Galactose
0.4
0.3
0.2
0.1
0.0
Abs(∞-0)
0
5
10
15
20
25
30
35
Concentration (w/w)

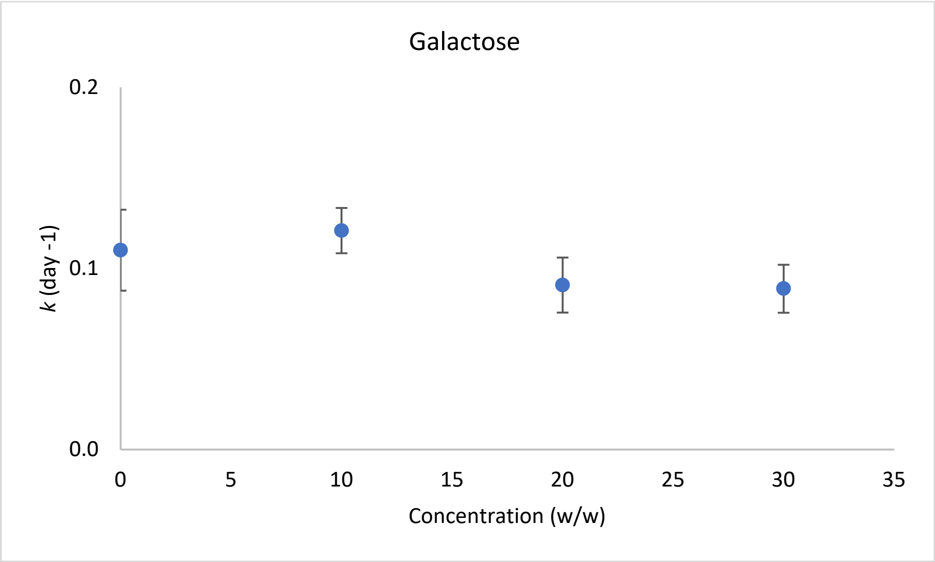

Galactose
0.2
0.1
0.0
k (day -1)
0
5
10
15
20
25
30
35
Concentration (w/w)


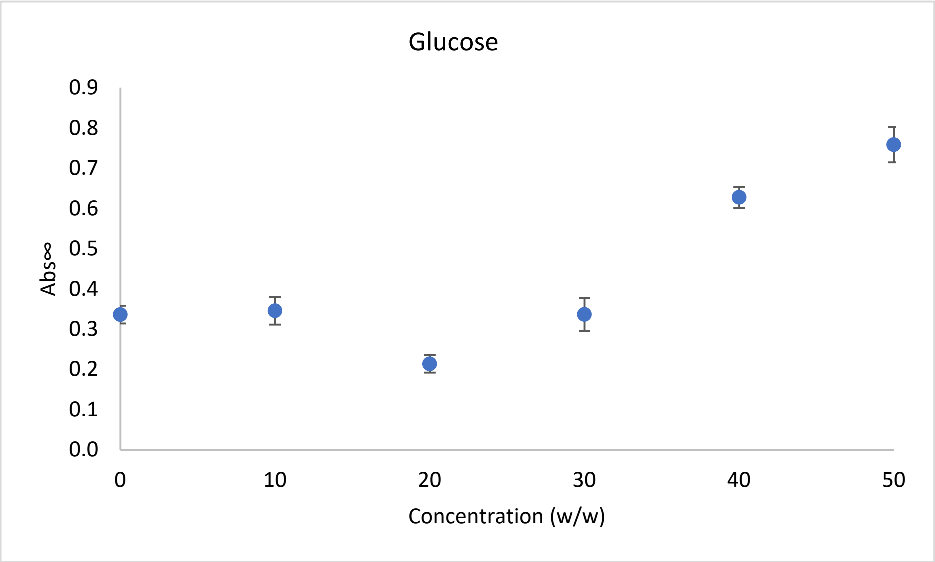

Glucose
0.9
0.8
0.7
0.6
0.5
0.4
0.3
0.2
0.1
0.0
Abs∞
0
10
20
30
40
50
Concentration (w/w)

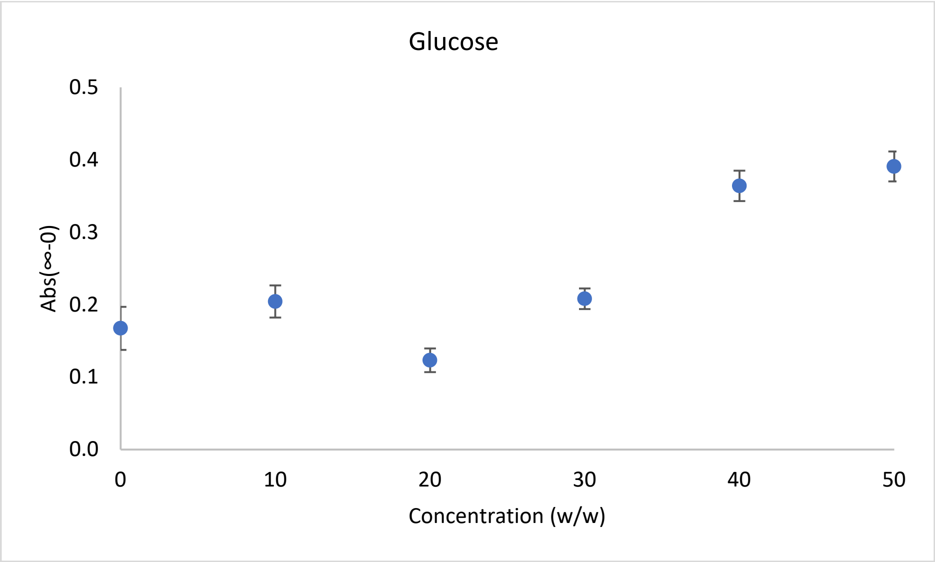
Glucose
0.5
0.4
0.3
0.2
0.1
0.0
Abs(∞-0)
0
10
20
30
40
50
Concentration (w/w)

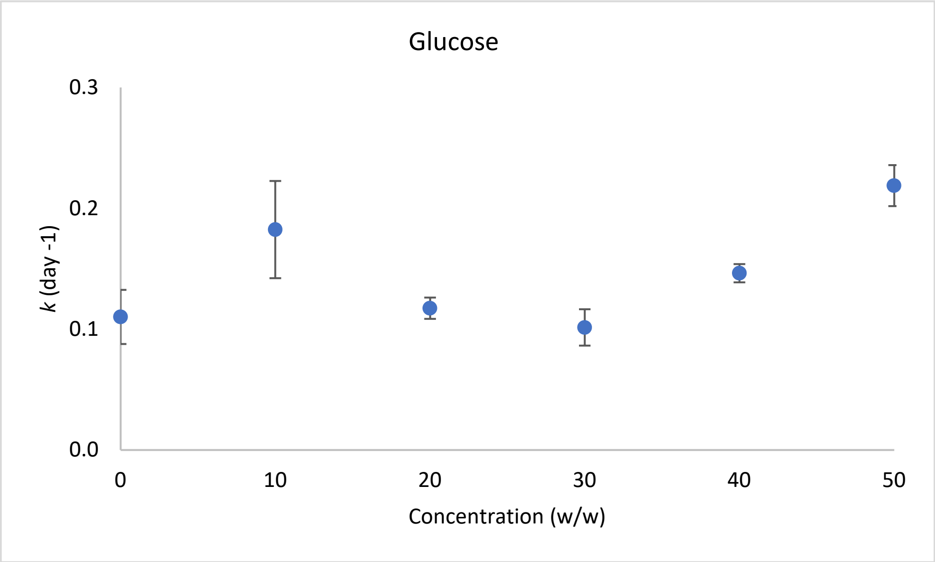
Glucose
0.3
0.2
0.1
0.0
k (day -1)
0
10
20
30
40
50
Concentration (w/w)

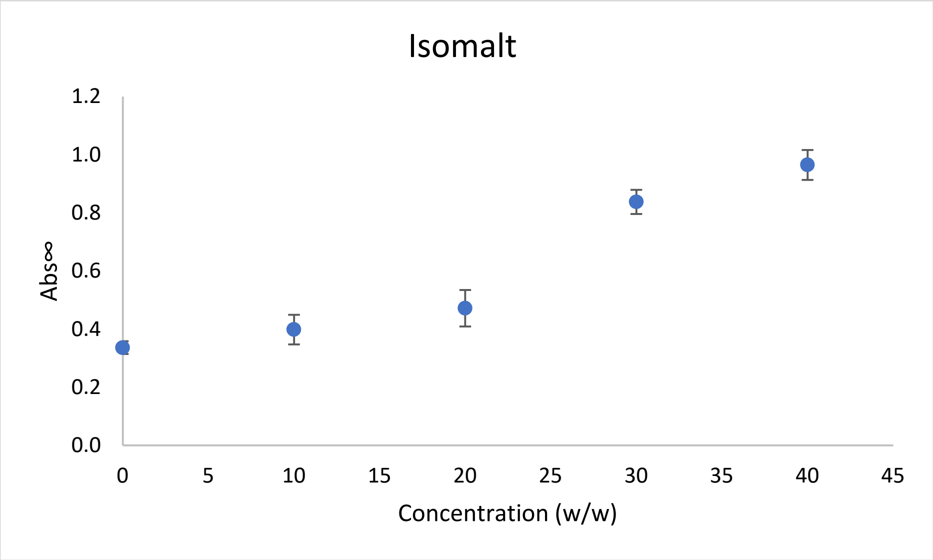
Isomalt
Abs∞
1.2
1.0
0.8
0.6
0.4
0.2
0.0
0
5
10
15
20
25
30
35
40
45
Concentration (w/w)

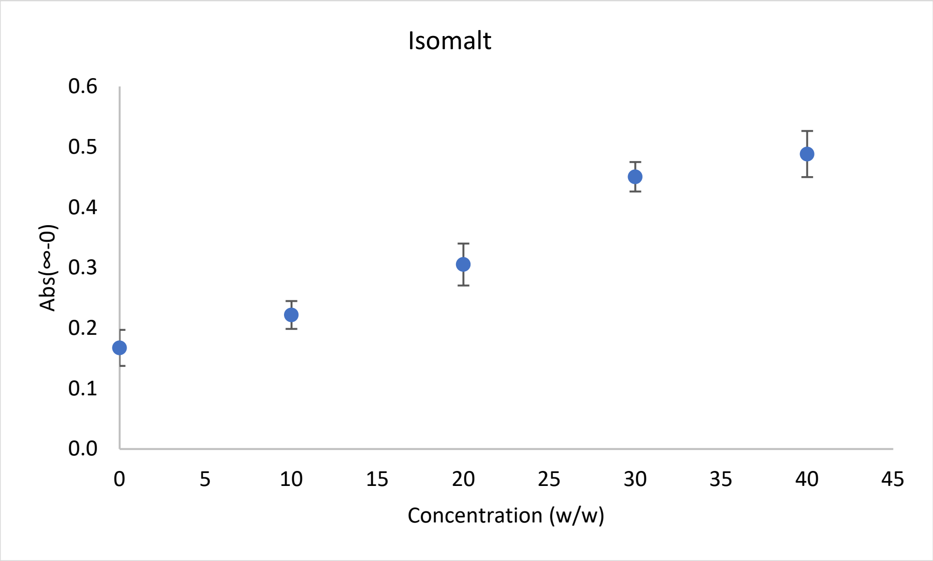
Isomalt
Abs(∞-0)
0.6
0.5
0.4
0.3
0.2
0.1
0.0
0
5
10
15
20
25
30
35
40
45
Concentration (w/w)

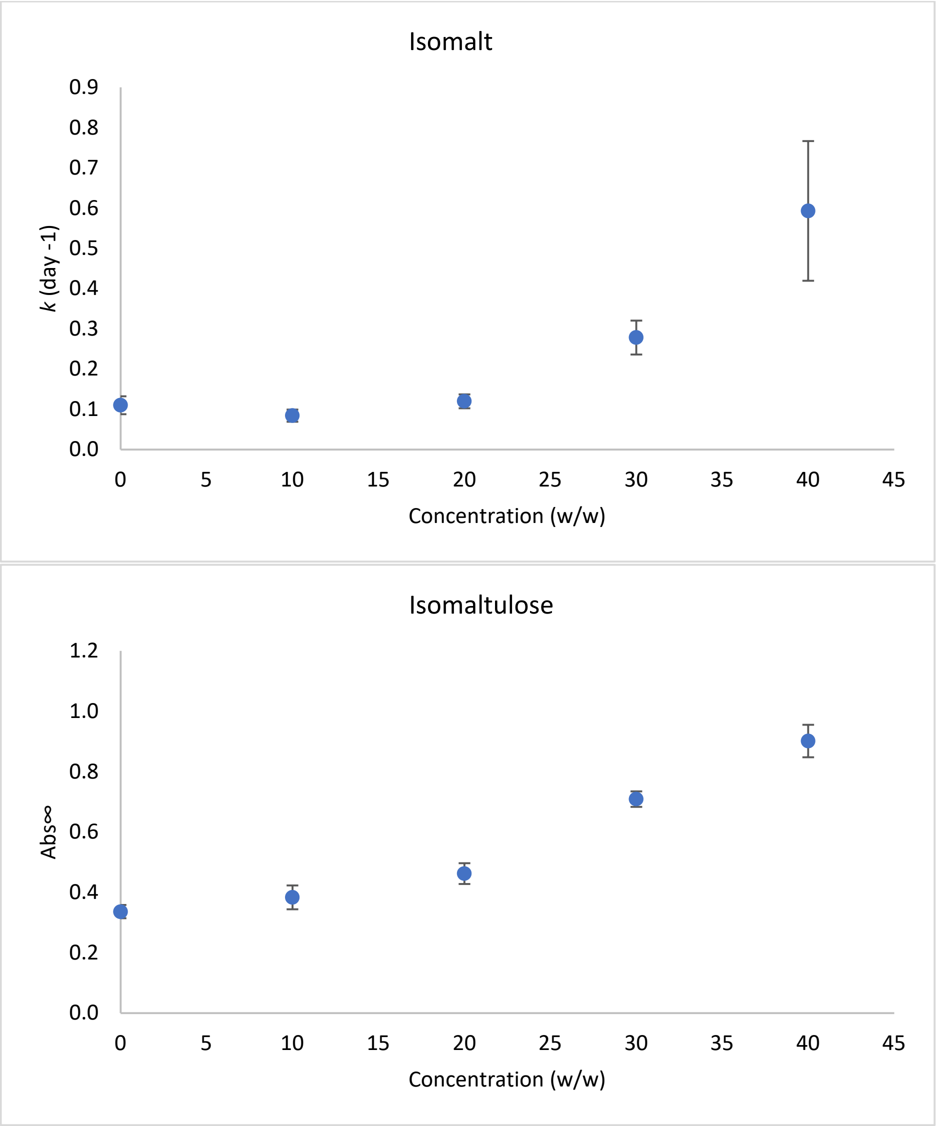

Isomalt
k (day -1)
0.0
0.1
0.2
0.3
0.4
0.5
0.6
0.7
0.8
0.9
0
5
10
15
20
25
30
35
40
45
Concentration (w/w)
Isomaltulose
Abs∞
0.0
0.2
0.4
0.6
0.8
1.0
1.2
0
5
10
15
20
25
30
35
40
45
Concentration (w/w)

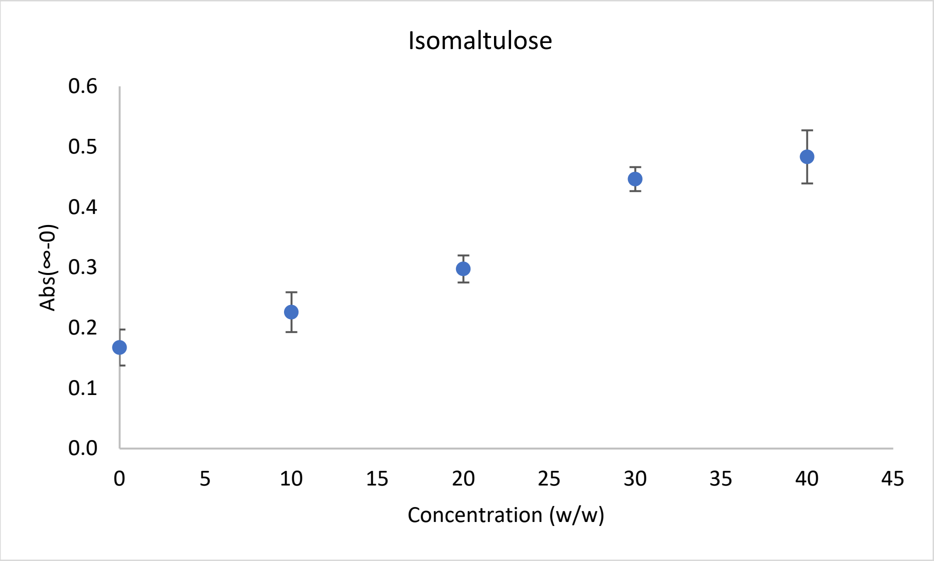
Isomaltulose
Abs(∞-0)
0.6
0.5
0.4
0.3
0.2
0.1
0.0
0 5 10 15 20 25 30 35 40 45
Concentration (w/w)

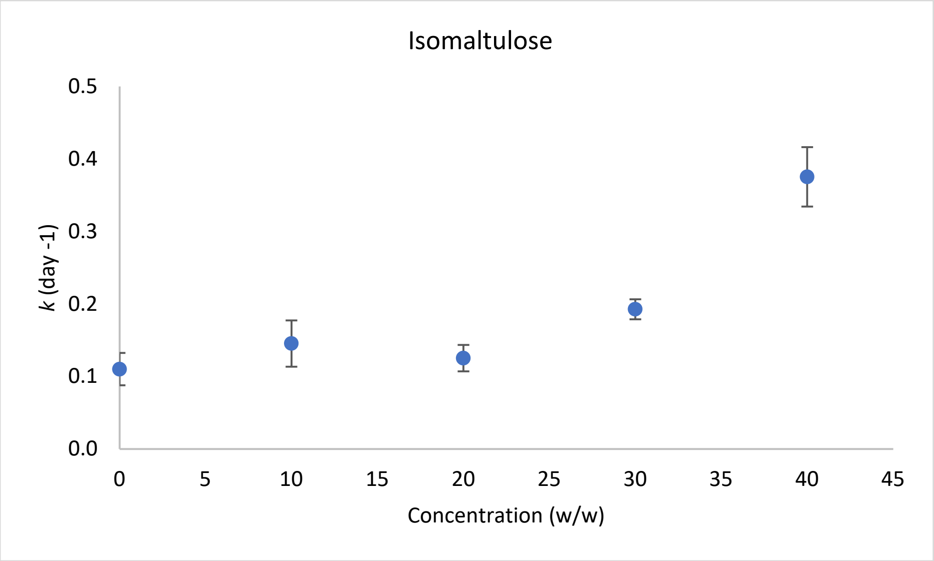
Isomaltulose
k (day -1)
0.5
0.4
0.3
0.2
0.1
0.0
0 5 10 15 20 25 30 35 40 45
Concentration (w/w)

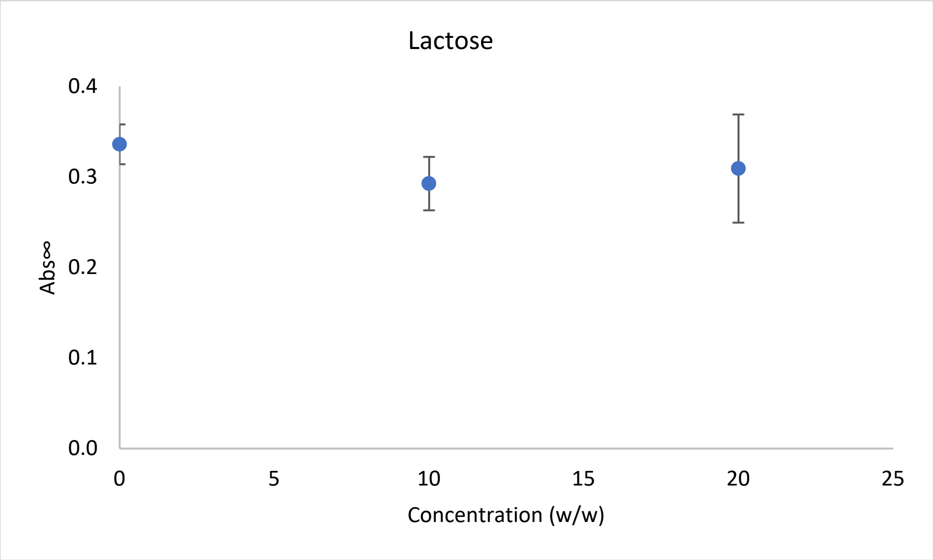
Lactose
0.4
0.3
0.2
0.1
0.0
Abs∞
0
5
10
15
20
25
Concentration (w/w)

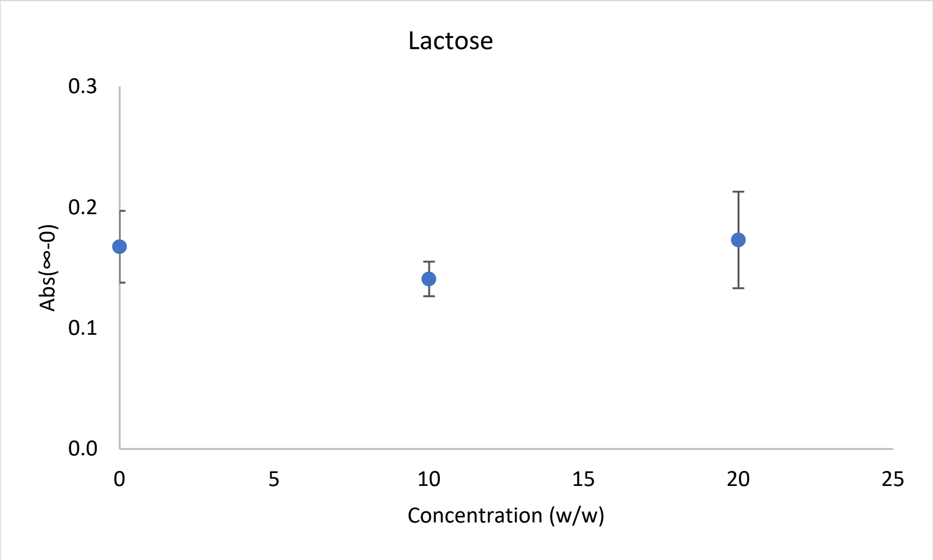
Lactose
0.3
0.2
0.1
0.0
Abs(∞-0)
0
5
10
15
20
25
Concentration (w/w)

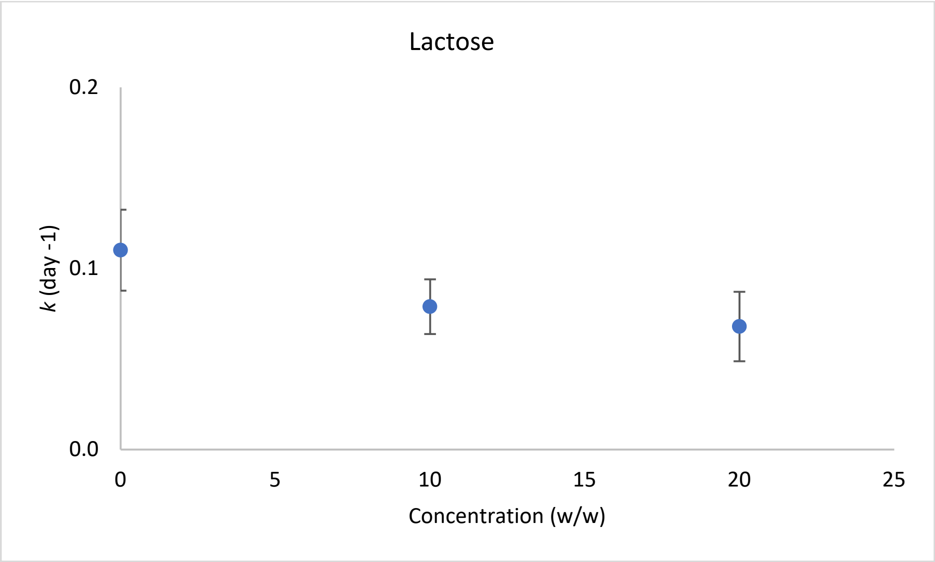
Lactose
0.2
0.1
0.0
k (day -1)
0
5
10
15
20
25
Concentration (w/w)

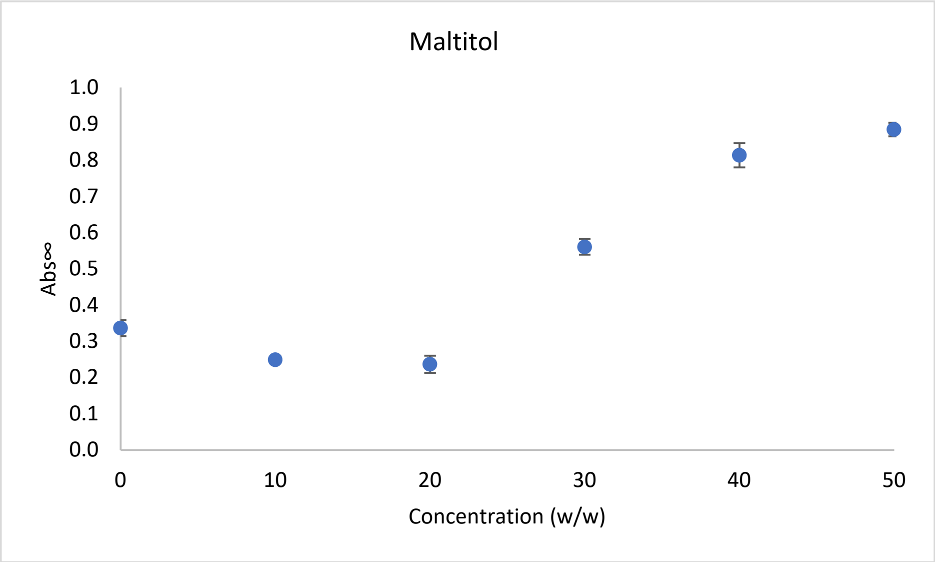
Maltitol
1.0
0.9
0.8
0.7
0.6
0.5
0.4
0.3
0.2
0.1
0.0
Abs∞
0
10
20
30
40
50
Concentration (w/w)

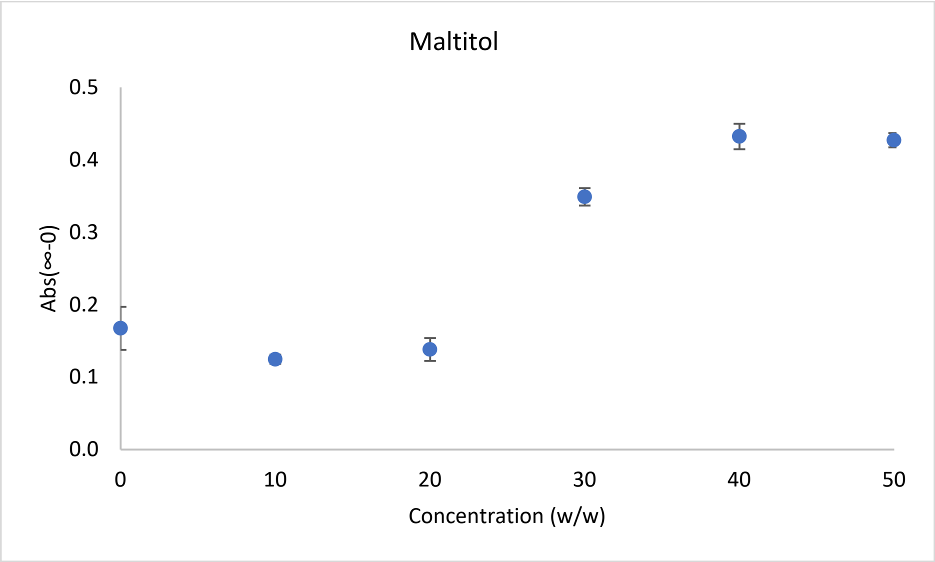
Maltitol
Abs(∞-0)
0.0
0.1
0.2
0.3
0.4
0.5
0
10
20
30
40
50
Concentration (w/w)

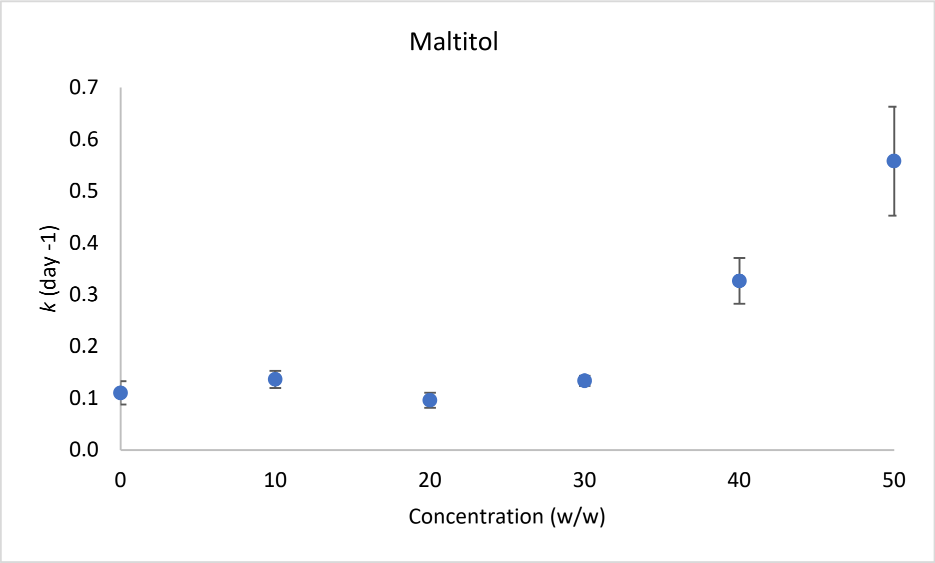
Maltitol
k (day -1)
0.0
0.1
0.2
0.3
0.4
0.5
0.6
0.7
0
10
20
30
40
50
Concentration (w/w)

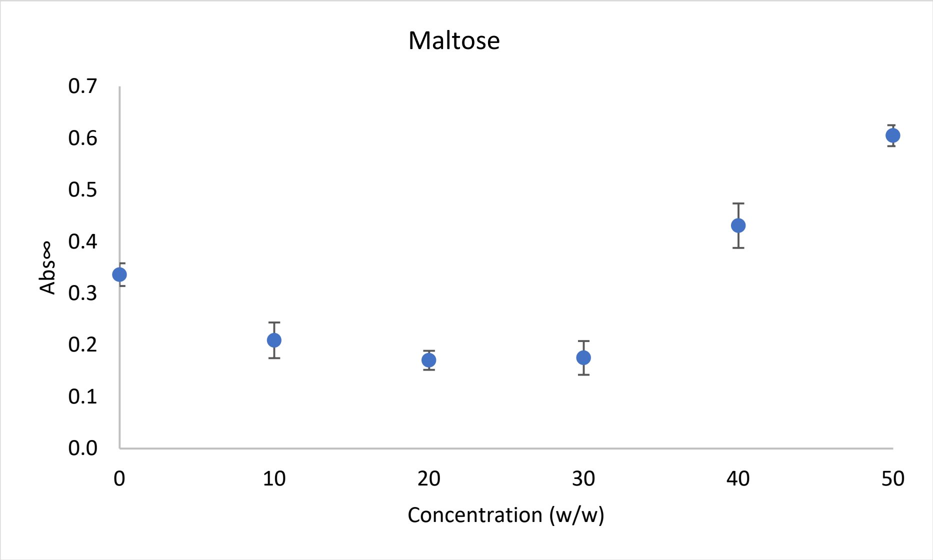
Maltose
Abs∞
0.7
0.6
0.5
0.4
0.3
0.2
0.1
0.0
0
10
20
30
40
50
Concentration (w/w)

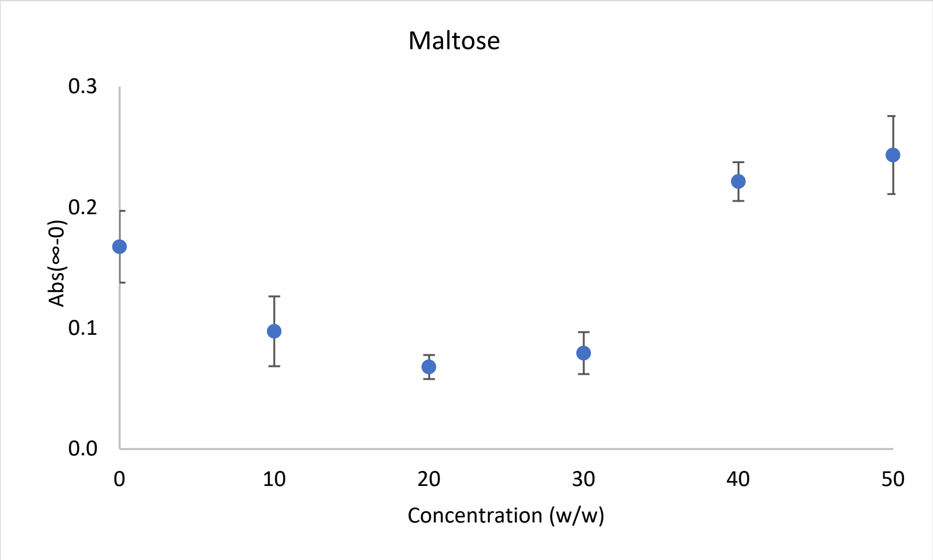
Maltose
Abs(∞-0)
0.3
0.2
0.1
0.0
0
10
20
30
40
50
Concentration (w/w)

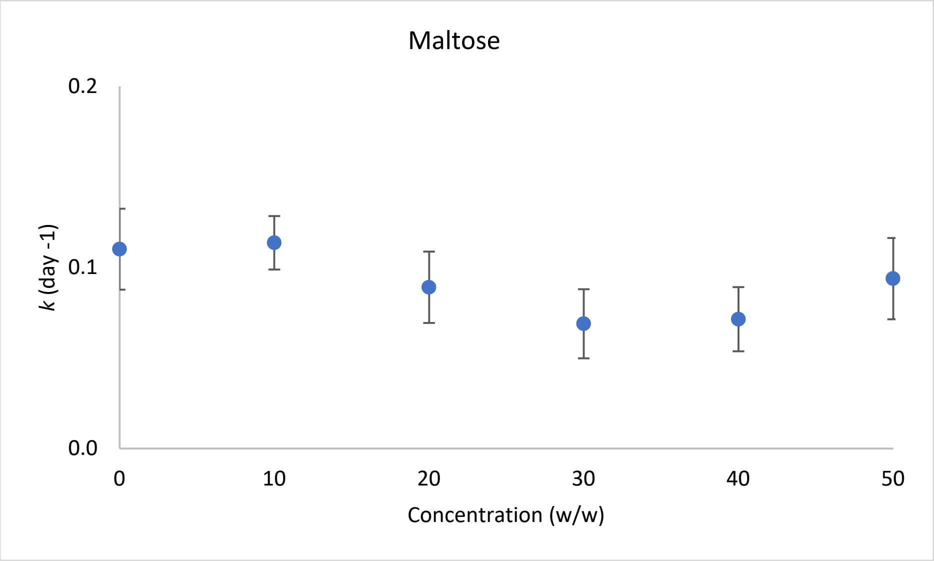
Maltose
0.2
0.1
0.0
k (day -1)
0
10
20
30
40
50
Concentration (w/w)

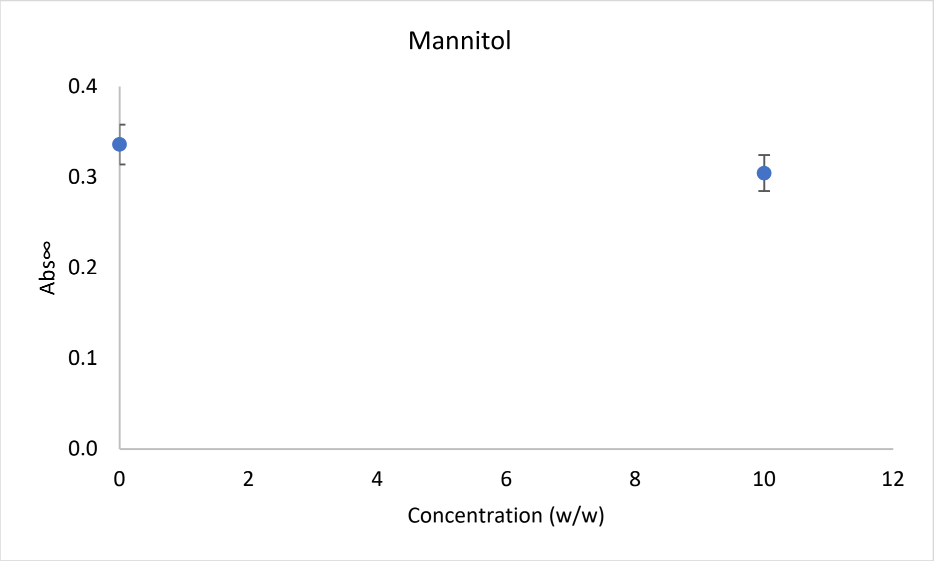
Mannitol
0.4
0.3
0.2
0.1
0.0
Abs∞
0
2
4
6
8
10
12
Concentration (w/w)

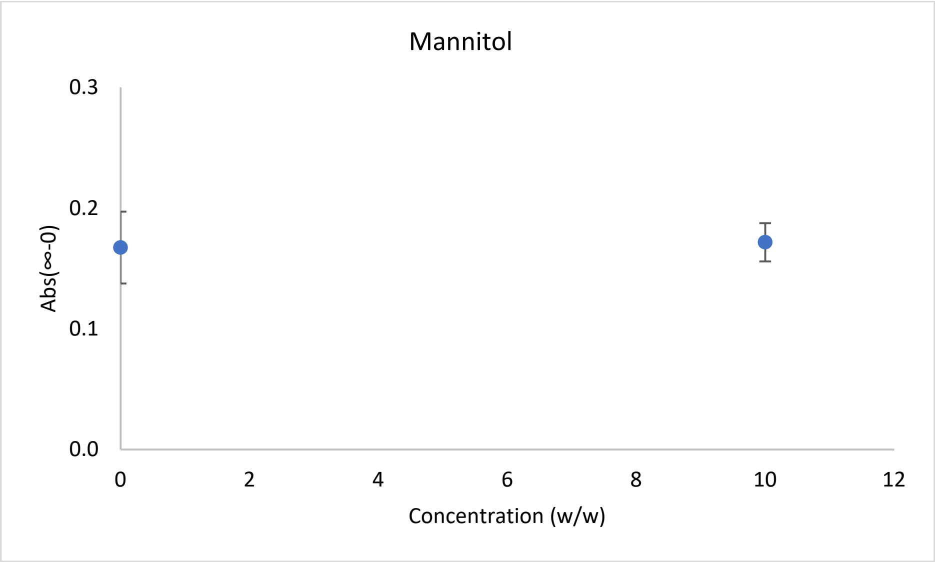
Mannitol
0.3
0.2
0.1
0.0
Abs(∞-0)
0
2
4
6
8
10
12
Concentration (w/w)

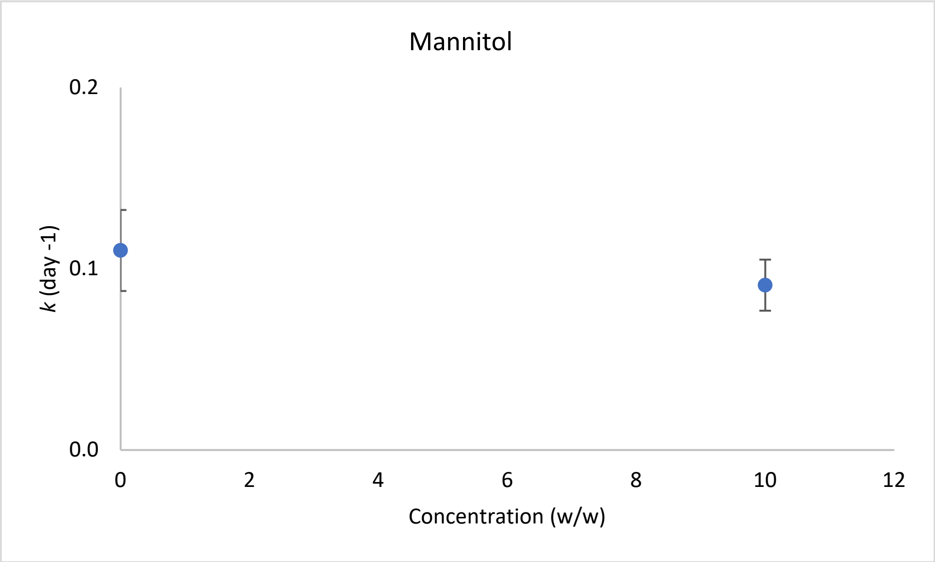
Mannitol
0.2
0.1
0.0
k (day -1)
0
2
4
6
8
10
12
Concentration (w/w)

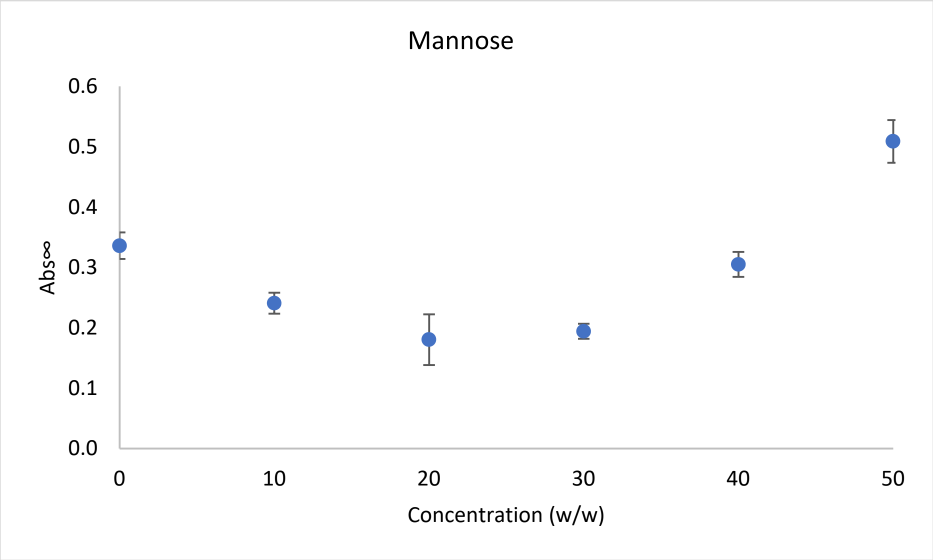
Mannose
0.6
0.5
0.4
0.3
0.2
0.1
0.0
Abs∞
0
10
20
30
40
50
Concentration (w/w)

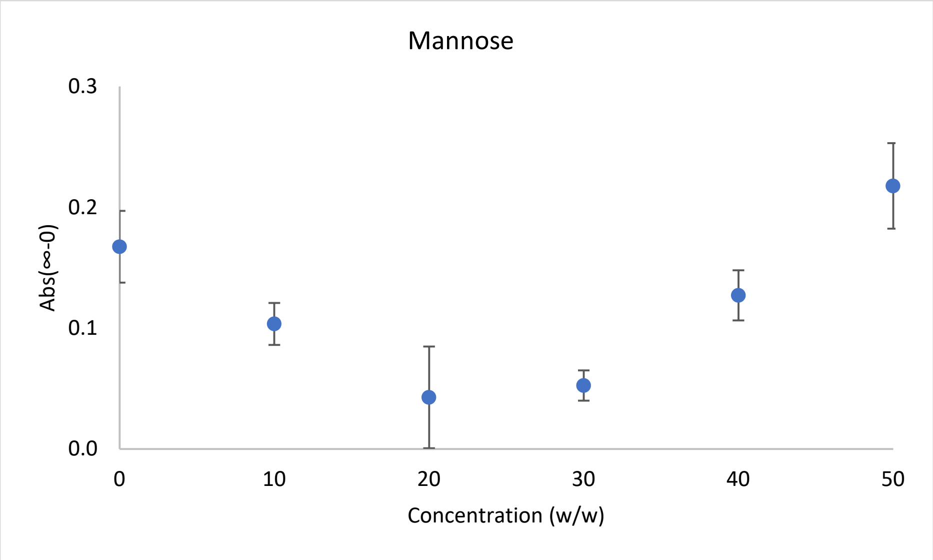
Mannose
0.3
0.2
0.1
0.0
Abs(∞-0)
0
10
20
30
40
50
Concentration (w/w)

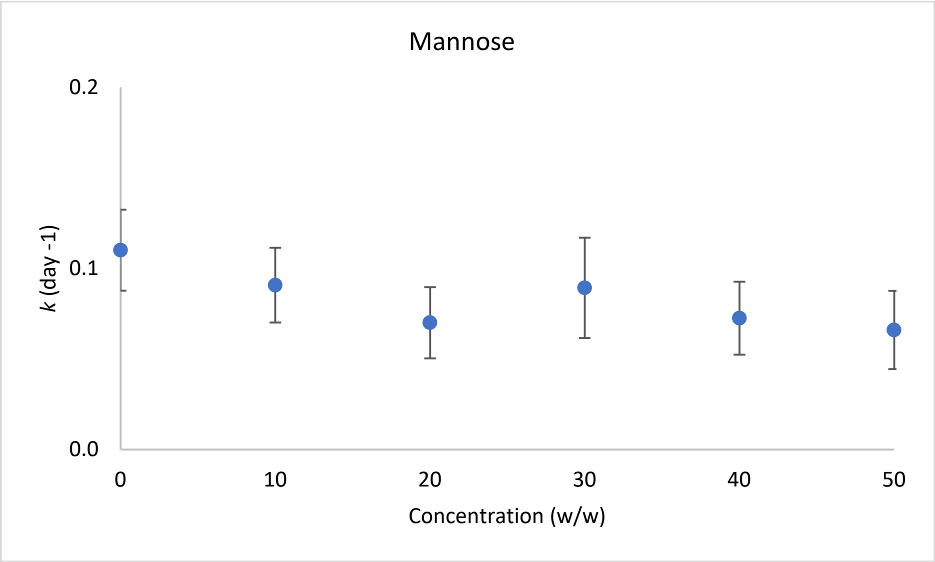
Mannose
0.2
0.1
0.0
k (day -1)
0
10
20
30
40
50
Concentration (w/w)

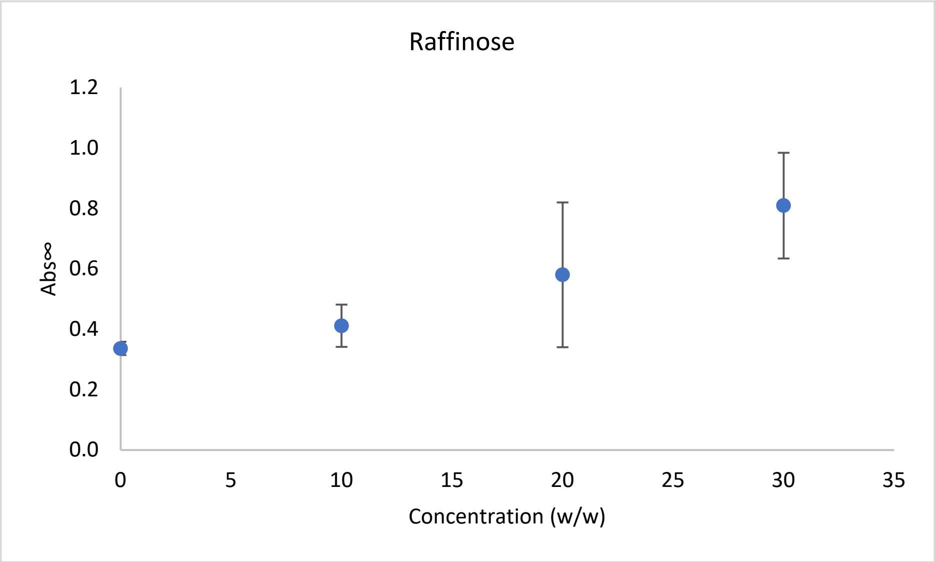
Raffinose
1.2
1.0
0.8
0.6
0.4
0.2
0.0
Abs∞
0
5
10
15
20
25
30
35
Concentration (w/w)

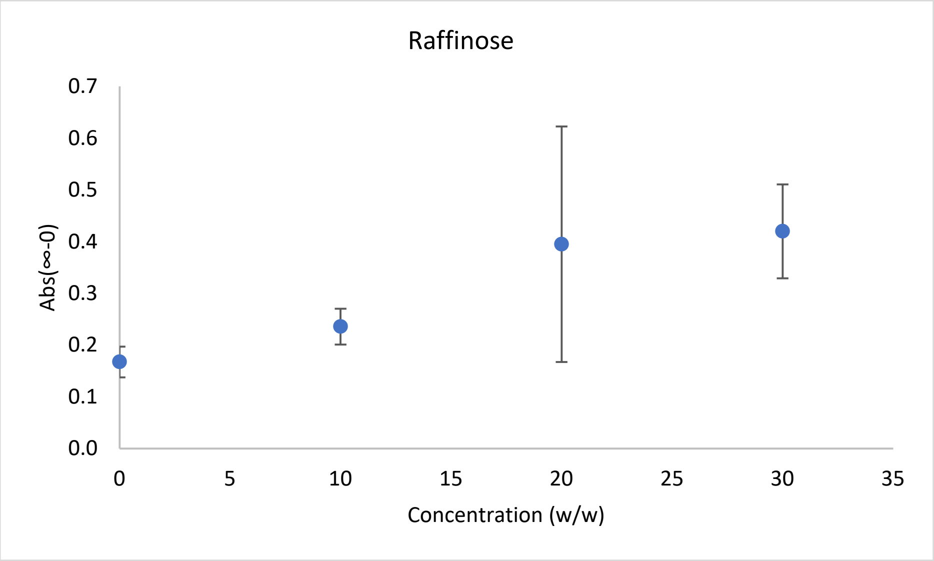
Raffinose
Abs(∞-0)
0.0
0.1
0.2
0.3
0.4
0.5
0.6
0.7
0
5
10
15
20
25
30
35
Concentration (w/w)

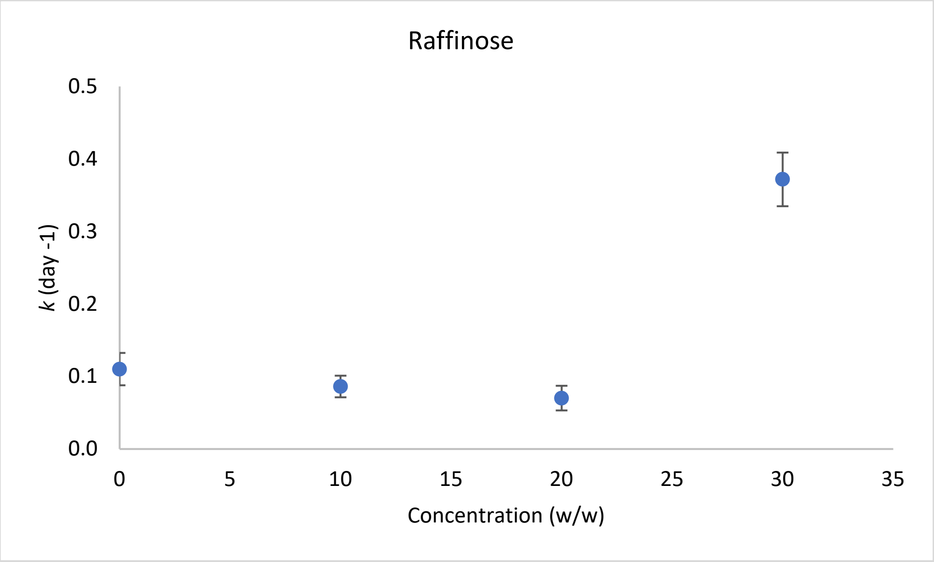
Raffinose
k (day -1)
0.0
0.1
0.2
0.3
0.4
0.5
0
5
10
15
20
25
30
35
Concentration (w/w)

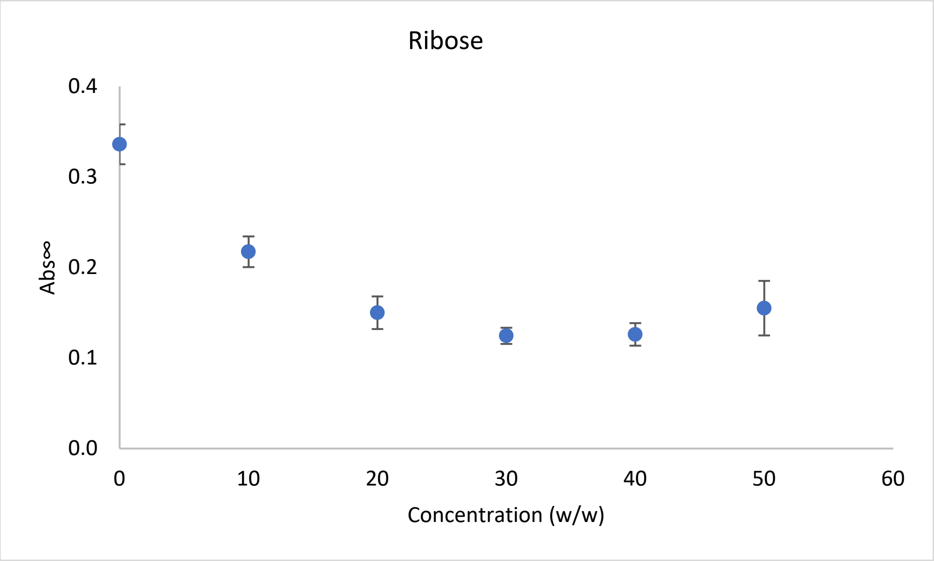
Ribose
0.4
0.3
0.2
0.1
0.0
Abs∞
0
10
20
30
40
50
60
Concentration (w/w)

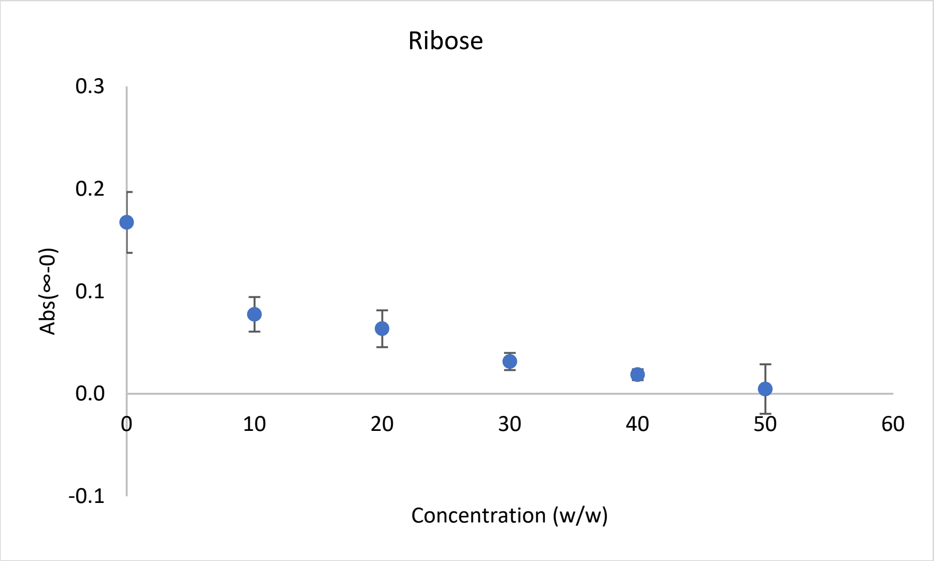
Ribose
0.3
0.2
0.1
0.0
-0.1
Abs(∞-0)
0
10
20
30
40
50
60
Concentration (w/w)

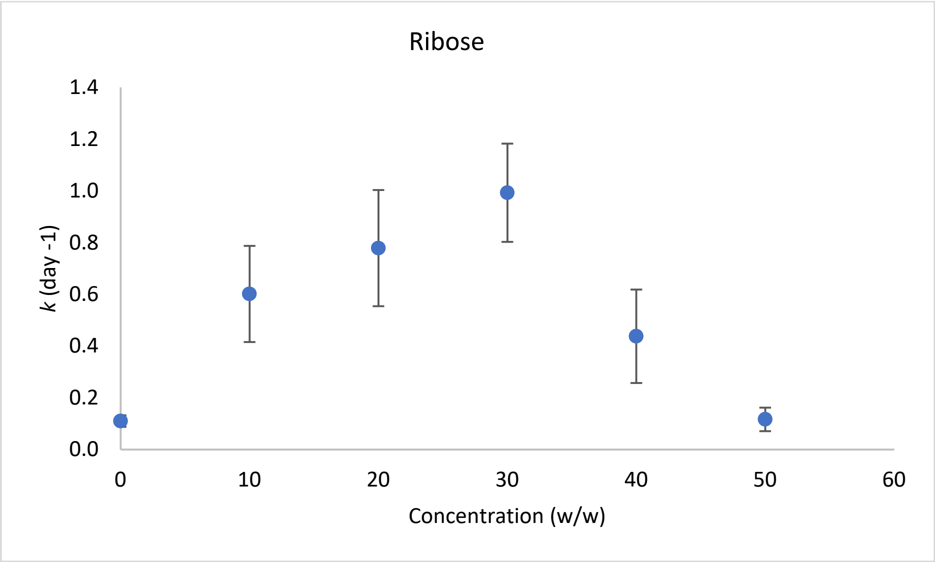
Ribose
1.4
1.2
1.0
0.8
0.6
0.4
0.2
0.0
k (day -1)
0
10
20
30
40
50
60
Concentration (w/w)

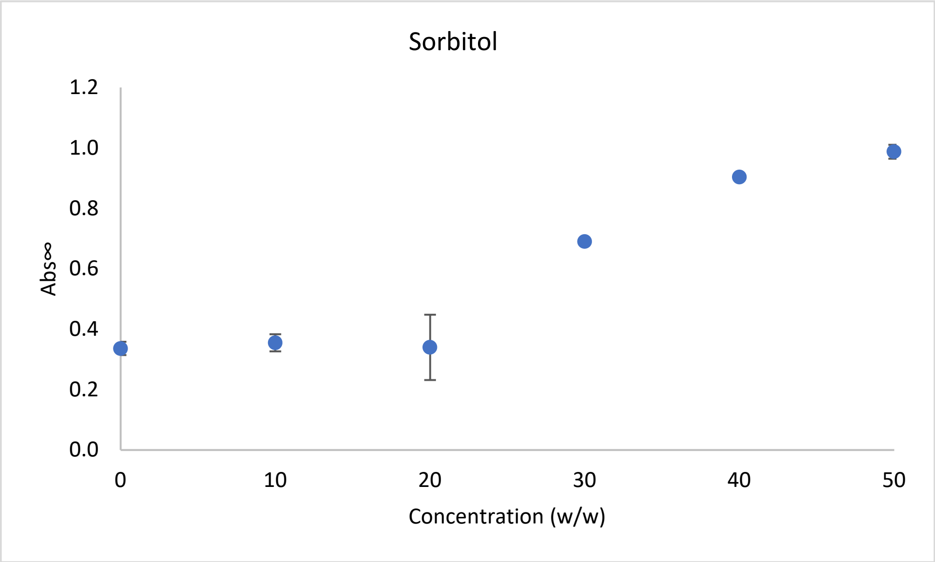
Sorbitol
1.2
1.0
0.8
0.6
0.4
0.2
0.0
Abs∞
0
10
20
30
40
50
Concentration (w/w)

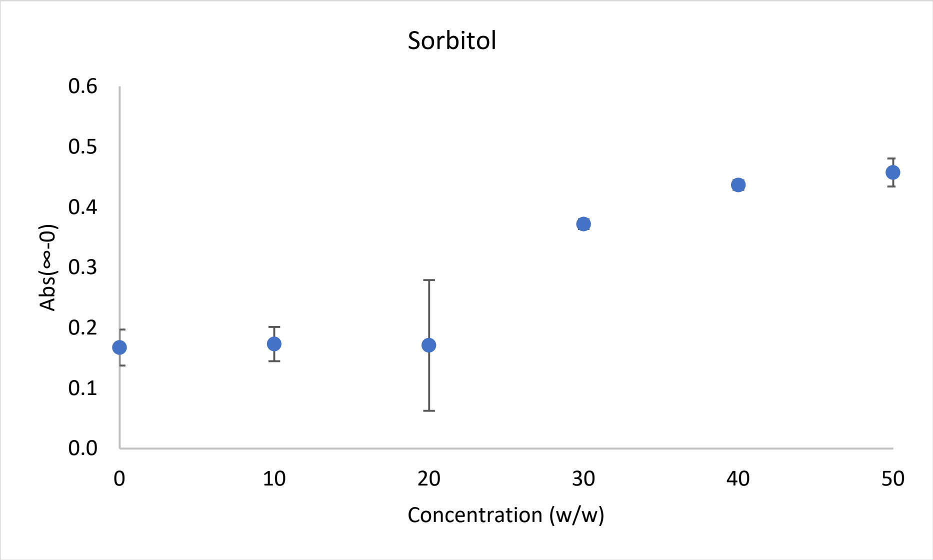
Sorbitol
Abs(∞-0)
0.0
0.1
0.2
0.3
0.4
0.5
0.6
0
10
20
30
40
50
Concentration (w/w)

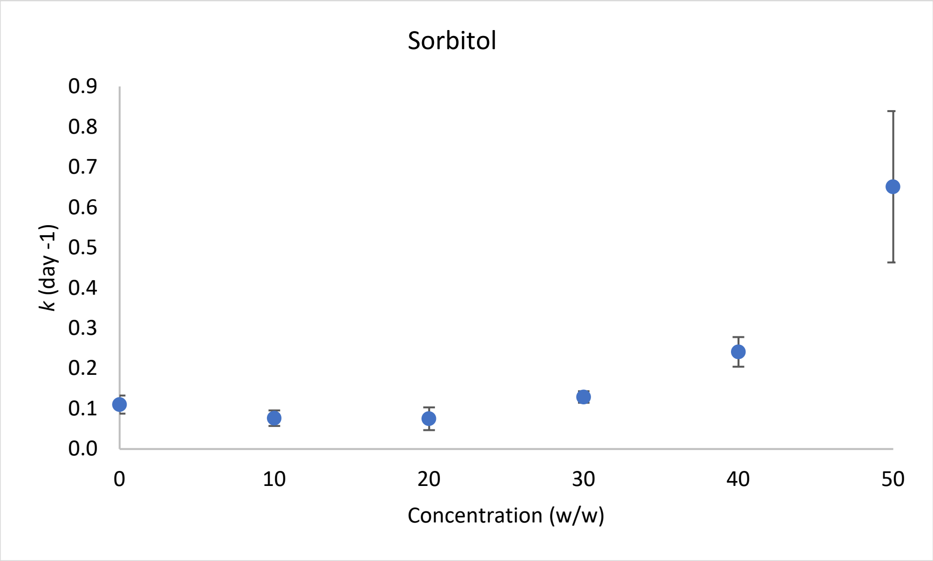
Sorbitol
k (day -1)
0.0
0.1
0.2
0.3
0.4
0.5
0.6
0.7
0.8
0.9
0
10
20
30
40
50
Concentration (w/w)

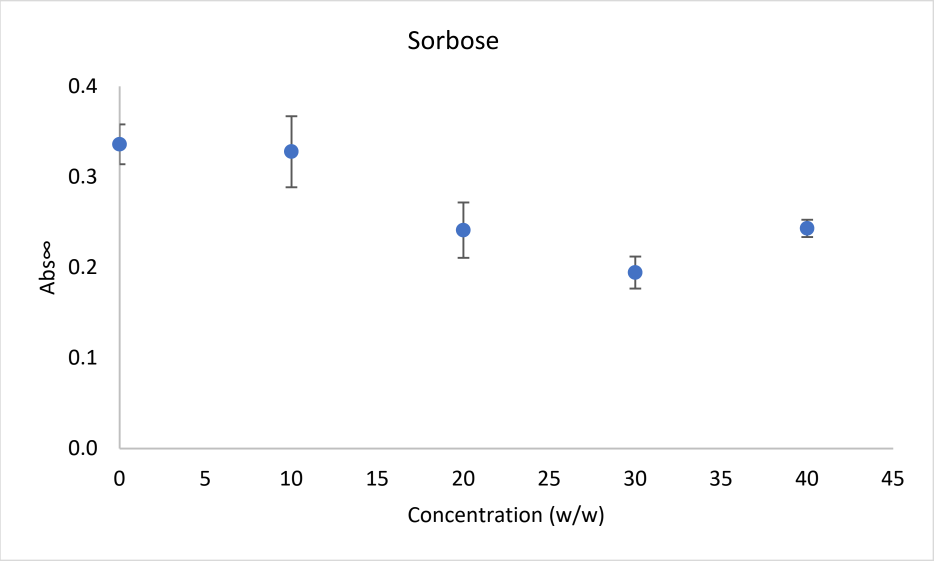
Sorbose
0.4
0.3
0.2
0.1
0.0
Abs∞
0
5
10
15
20
25
30
35
40
45
Concentration (w/w)

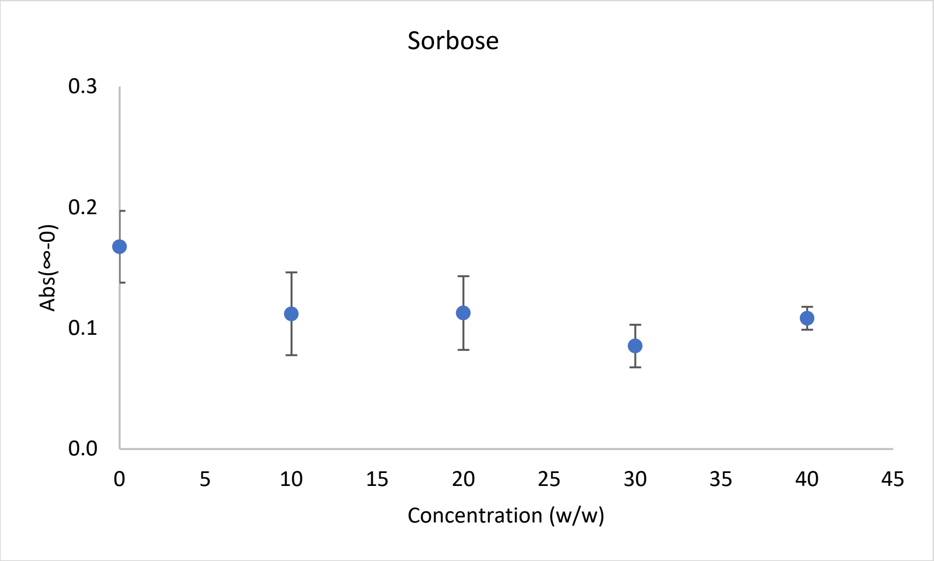
Sorbose
0.3
0.2
0.1
0.0
Abs(∞-0)
0
5
10
15
20
25
30
35
40
45
Concentration (w/w)

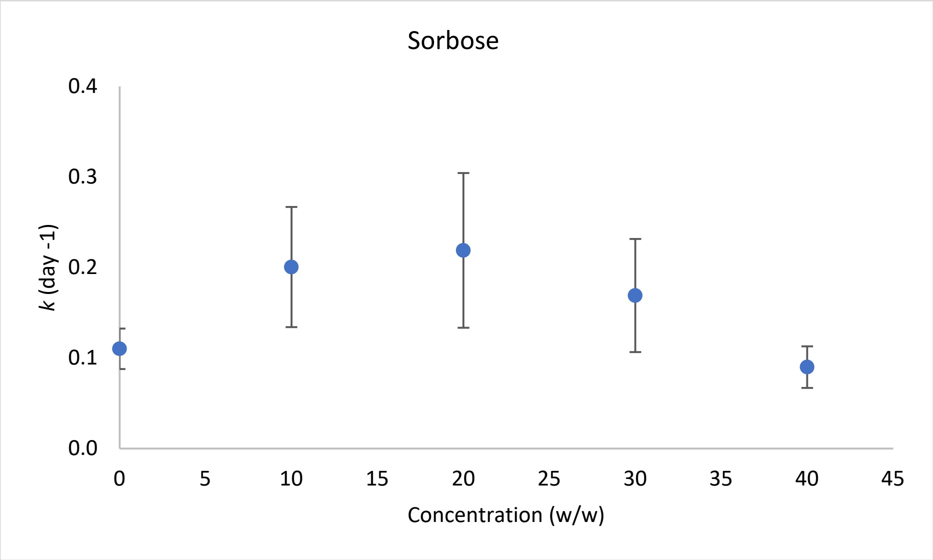

Sorbose
0.4
0.3
0.2
0.1
0.0
k (day -1)
0
5
10
15
20
25
30
35
40
45
Concentration (w/w)


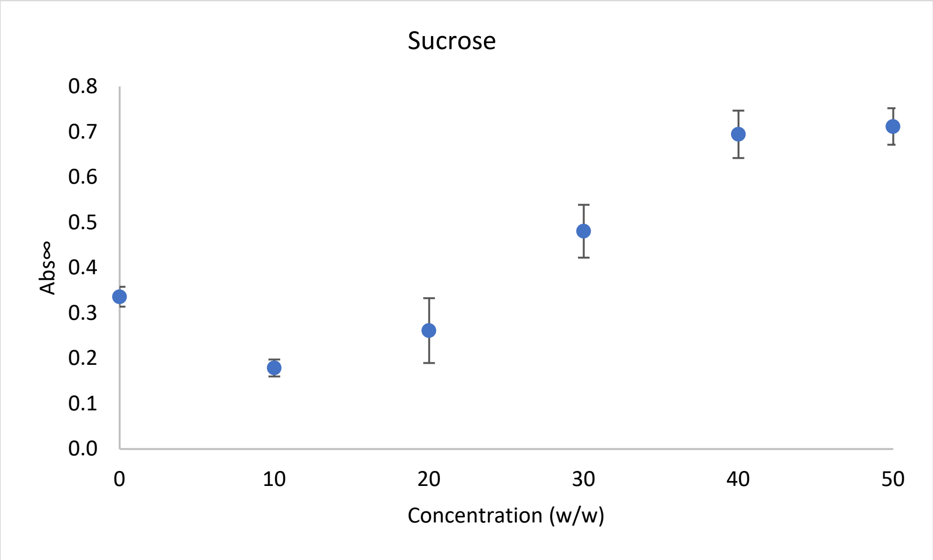

Sucrose
0.8
0.7
0.6
0.5
0.4
0.3
0.2
0.1
0.0
Abs∞
0
10
20
30
40
50
Concentration (w/w)

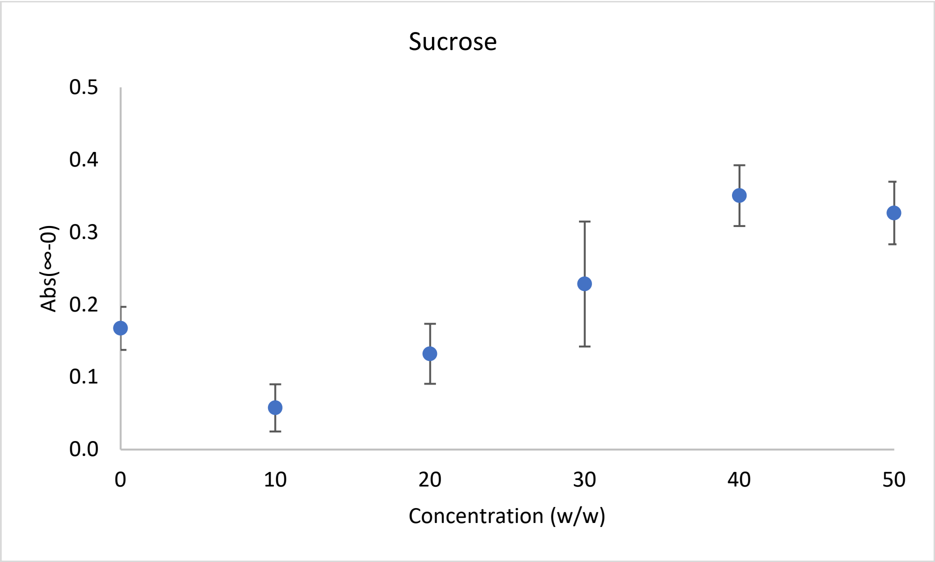

Sucrose
0.5
0.4
0.3
0.2
0.1
0.0
Abs(∞-0)
0
10
20
30
40
50
Concentration (w/w)


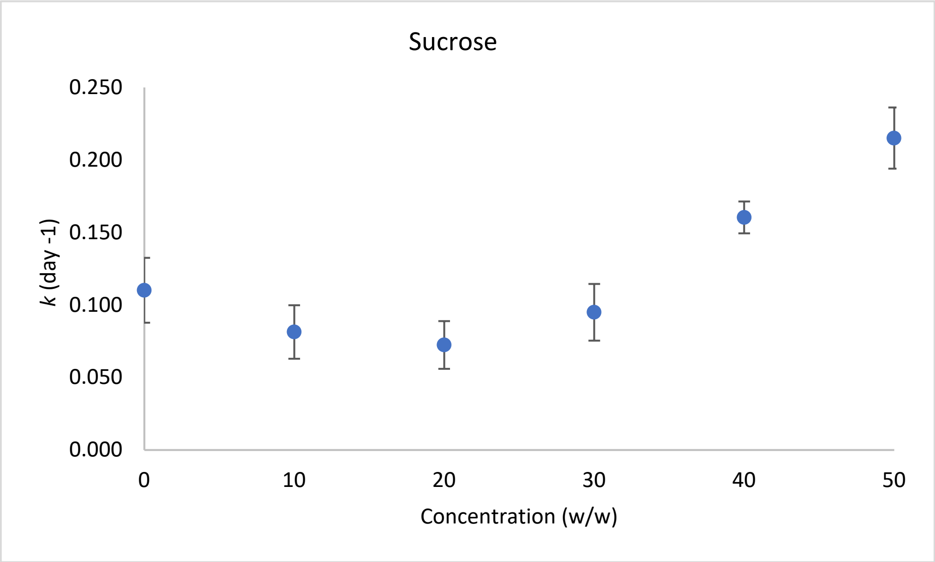

Sucrose
0.250
0.200
0.150
0.100
0.050
0.000
k (day -1)
0
10
20
30
40
50
Concentration (w/w)

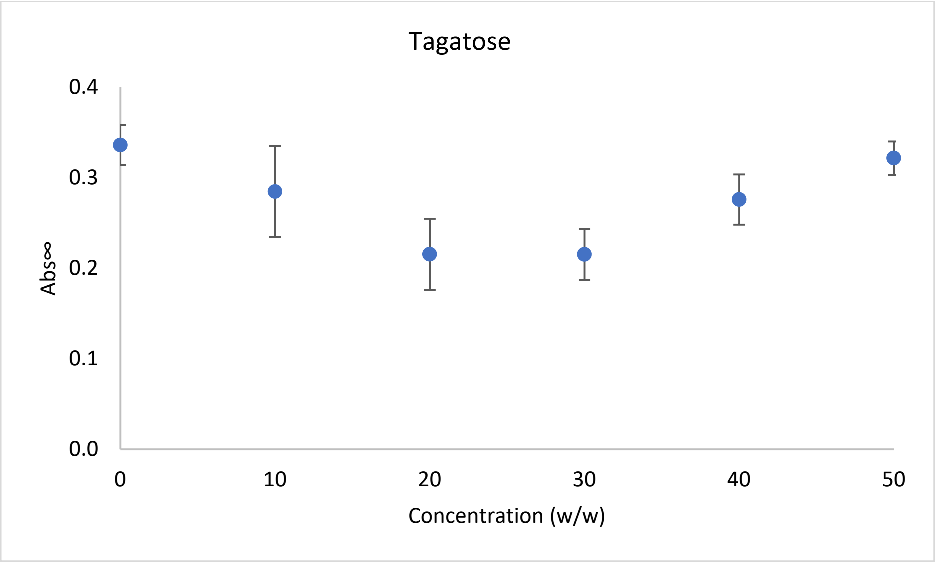
Tagatose
0.4
0.3
0.2
0.1
0.0
Abs∞
0
10
20
30
40
50
Concentration (w/w)

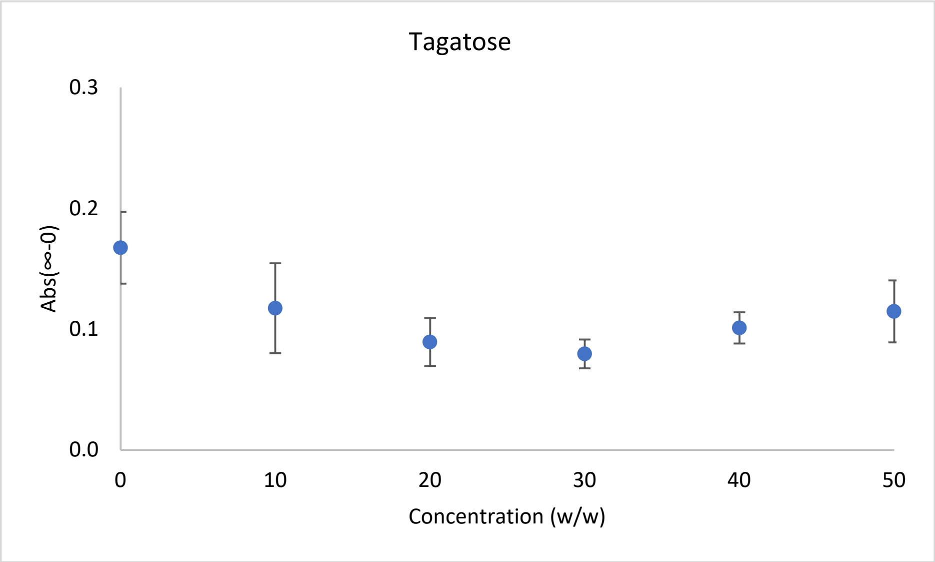
Tagatose
0.3
0.2
0.1
0.0
Abs(∞-0)
0
10
20
30
40
50
Concentration (w/w)

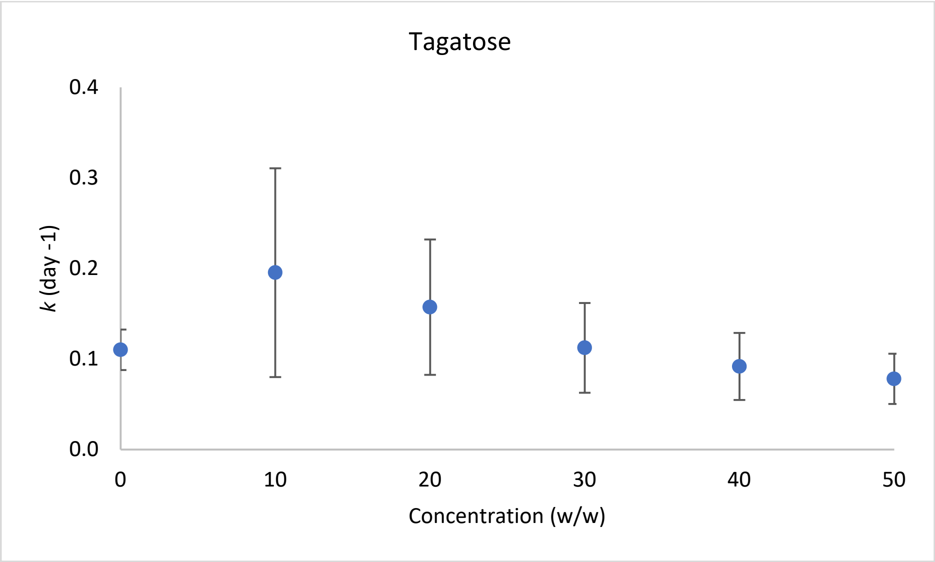
Tagatose
k (day -1)
0.4
0.3
0.2
0.1
0.0
0
10
20
30
40
50
Concentration (w/w)

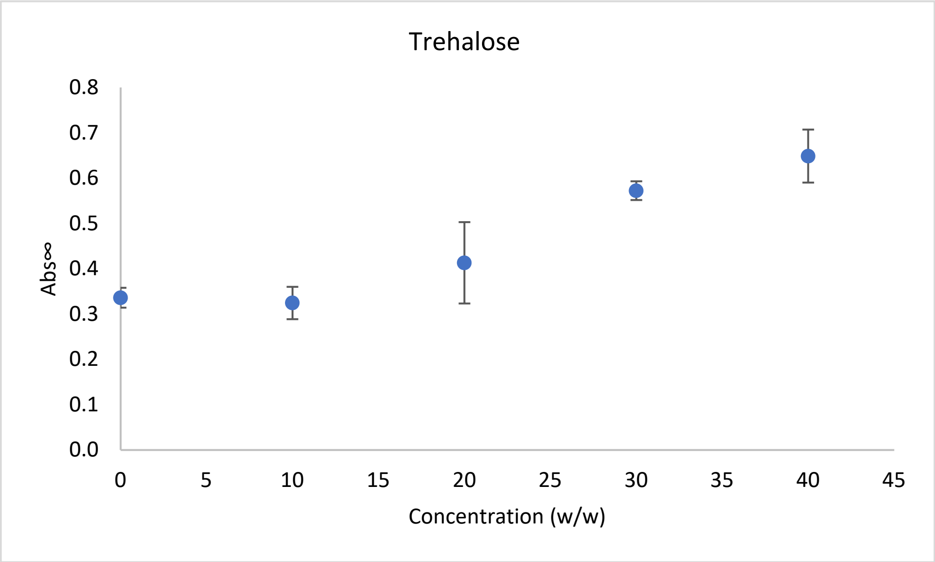
Trehalose
Abs∞
0.8
0.7
0.6
0.5
0.4
0.3
0.2
0.1
0.0
0
5
10
15
20
25
30
35
40
45
Concentration (w/w)

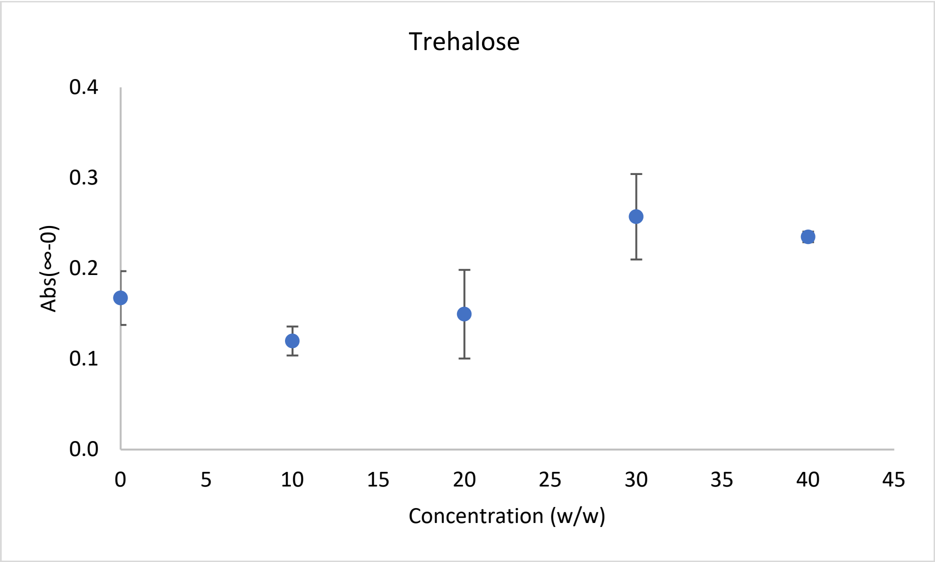
Trehalose
0.4
0.3
0.2
0.1
0.0
Abs(∞-0)
0
5
10
15
20
25
30
35
40
45
Concentration (w/w)

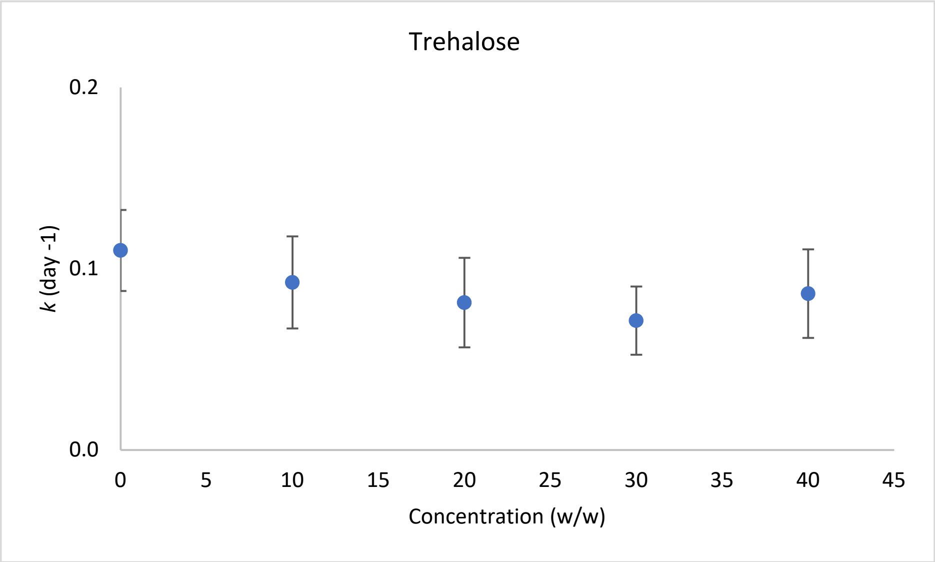
Trehalose
0.2
0.1
0.0
k (day -1)
0
5
10
15
20
25
30
35
40
45
Concentration (w/w)

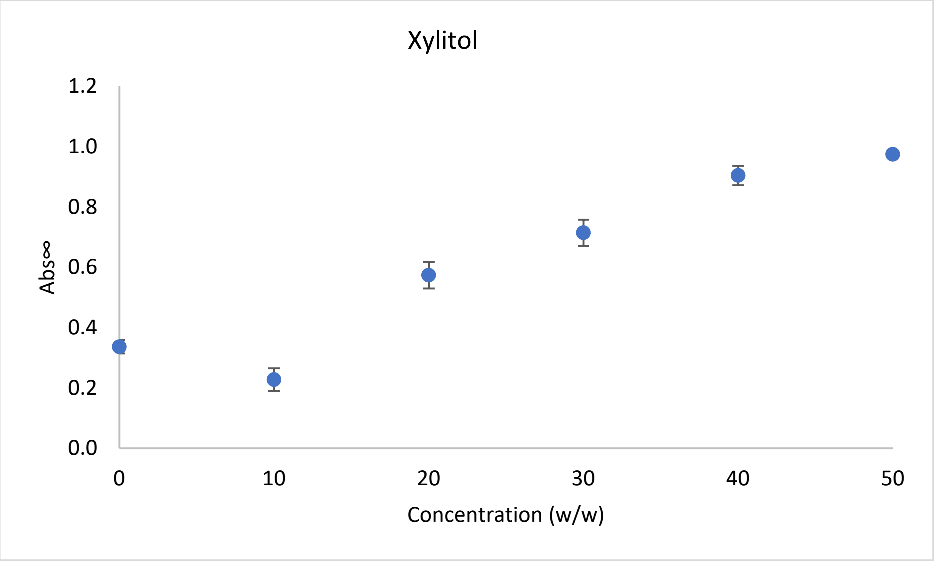
Xylitol
1.2
1.0
0.8
0.6
0.4
0.2
0.0
Abs∞
0
10
20
30
40
50
Concentration (w/w)

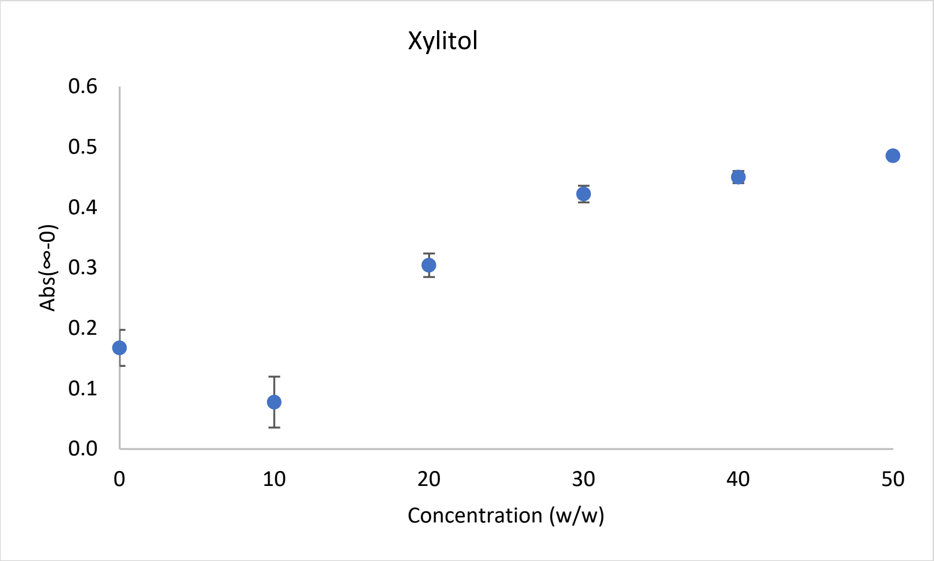
Xylitol
0.6
0.5
0.4
0.3
0.2
0.1
0.0
Abs(∞-0)
0
10
20
30
40
50
Concentration (w/w)

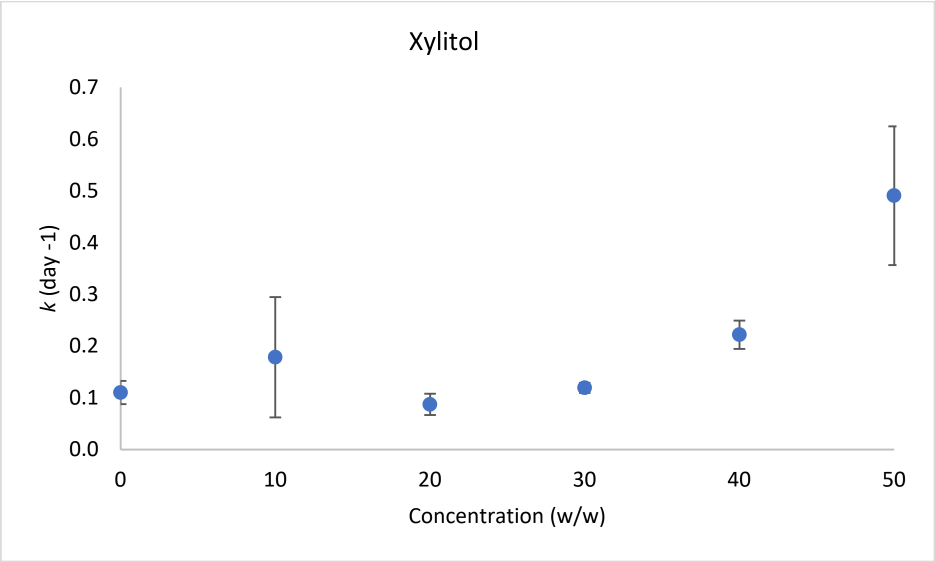
Xylitol
0.7
0.6
0.5
0.4
0.3
0.2
0.1
0.0
k (day -1)
0
10
20
30
40
50
Concentration (w/w)

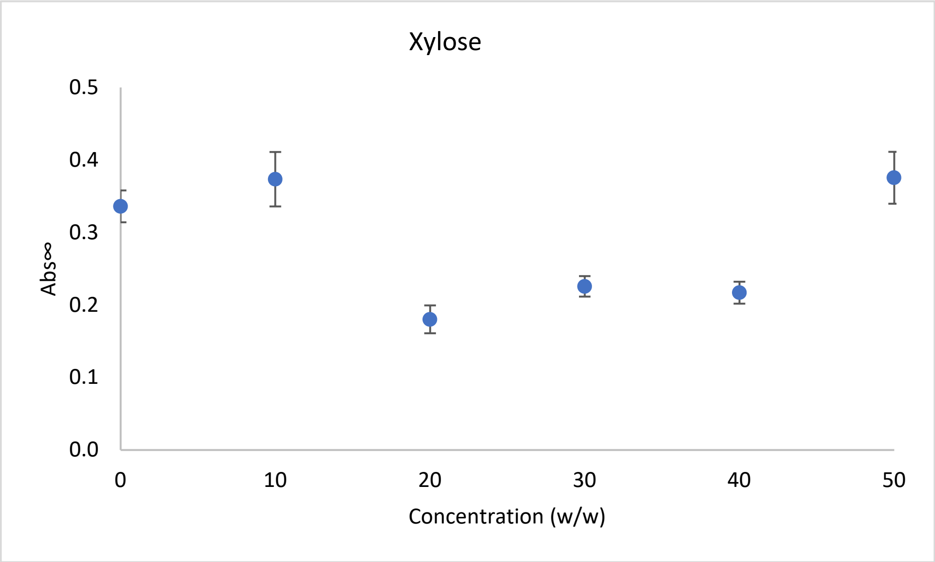
Xylose
0.5
0.4
0.3
0.2
0.1
0.0
Abs∞
0
10
20
30
40
50
Concentration (w/w)

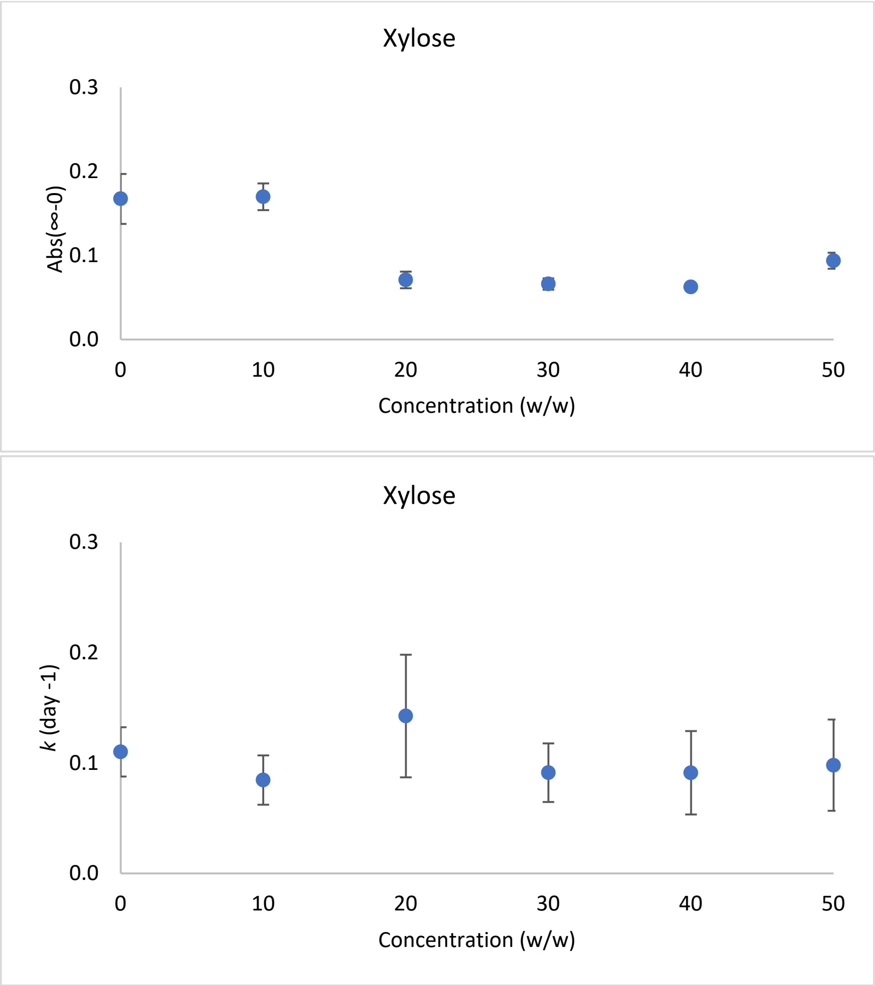


**Figure S1.** Plots of 620 nm absorbances after 28 days ($Abs_{\infty}$) with one standard deviation, changes of 620 nm absorbances from day 0 to day 28 ($Abs_{(\infty\text{-}0)}$) with one standard deviation, and the calculated Avrami rate constants (*k*) with 95% confidence intervals that were grouped by sweetener concentrations within sweeteners.

## References


1. Goldberg, R.N.; Tewari, Y.B. Thermodynamic and transport properties of carbohydrates and their monophosphates: The pentoses and hexoses. *J. Phys. Chem. Ref. Data* **1989**, *18*, 809–880. https://doi.org/10.1063/1.555831.
2. Angyal, S.; Bethell, G. Conformational analysis in carbohydrate chemistry. III. The $^{13}$C NMR spectra of the hexuloses. *Aust. J. Chem.* **1976**, *29*, 1249–1265. https://doi.org/10.1071/CH9761249.
3. Bell, A.F.; Hecht, L.; Barron, L.D. Disaccharide solution stereochemistry from vibrational Raman optical activity. *J. Am. Chem. Soc.* **1994**, *116*, 5155–5161. https://doi.org/10.1021/ja00091a019.
4. Lichtenthaler, F.W.; Rönninger, S. $\alpha$-D-Glucopyranosyl-D-fructoses: Distribution of furanoid and pyranoid tautomers in water, dimethyl sulphoxide, and pyridine. Studies on ketoses. Part 4. *J. Chem. Soc. Perkin Trans. 2* **1990**, *8*, 1489–1497. https://doi.org/10.1039/P29900001489.
5. Fox, P.F.; Uniacke-Lowe, T.; McSweeney, P.L.H.; O'Mahony, J.A. Lactose. In *Dairy Chemistry and Biochemistry*, 2nd ed.; Springer: Berlin/Heidelberg, Germany, 2015; pp. 21–68.
6. Uedaira, H.; Ikura, M.; Uedaira, H. Natural-abundance oxygen-17 magnetic relaxation in aqueous solutions of carbohydrates. *Bull. Chem. Soc. Jpn.* **1989**, *62*, 1–4. https://doi.org/10.1246/bcsj.62.1.
7. Kajiwara, K.; Franks, F.; Echlin, P.; Greer, A.L. Structural and dynamic properties of crystalline and amorphous phases in raffinose-water mixtures. *Pharm. Res.* **1999**, *16*, 1441–1448. https://doi.org/10.1023/a:1018915512289.
8. Kawai, H.; Sakurai, M.; Inoue, Y.; Chûjô, R.; Kobayashi, S. Hydration of oligosaccharides: Anomalous hydration ability of trehalose. *Cryobiology* **1992**, *29*, 599–606. https://doi.org/10.1016/0011-2240(92)90064-9.
9. Shamil, S.; Birch, G.; Mathlouthi, M.; Clifford, M. Apparent molar volumes and tastes of molecules with more than one sapo-692 phore. Chem. Senses 1987, 12, 397-409, doi:https://doi.org/10.1093/chemse/12.2.397.
